# Nanomaterials for Direct Air Capture of CO_2_: Current State of the Art, Challenges and Future Perspectives

**DOI:** 10.3390/molecules30143048

**Published:** 2025-07-21

**Authors:** Cataldo Simari

**Affiliations:** 1 Department of Chemistry and Chemical Technology, University of Calabria, Via P. Bucci, 87036 Cosenza, Italy; cataldo.simari@unical.it; Tel.: +39-0984-493385; Fax: +39-0984-492044; 2 GISEL-Centro di Riferimento Nazionale per i Sistemi di Accumulo Elettrochimico di Energia, INSTM, Via G. Giusti 9, 50121 Firenze, Italy

**Keywords:** CO_2_ capture, direct air capture, atmospheric air, moisture swing adsorption, solid sorbents

## Abstract

Direct Air Capture (DAC) is emerging as a critical climate change mitigation strategy, offering a pathway to actively remove atmospheric CO_2_. This comprehensive review synthesizes advancements in DAC technologies, with a particular emphasis on the pivotal role of nanostructured solid sorbent materials. The work critically evaluates the characteristics, performance, and limitations of key nanomaterial classes, including metal–organic frameworks (MOFs), covalent organic frameworks (COFs), zeolites, amine-functionalized polymers, porous carbons, and layered double hydroxides (LDHs), alongside solid-supported ionic liquids, highlighting their varied CO_2_ uptake capacities, regeneration energy requirements, and crucial water sensitivities. Beyond traditional temperature/pressure swing adsorption, the review delves into innovative DAC methodologies such as Moisture Swing Adsorption (MSA), Electro Swing Adsorption (ESA), Passive DAC, and CO_2_-Binding Organic Liquids (CO_2_ BOLs), detailing their unique mechanisms and potential for reduced energy footprints. Despite significant progress, the widespread deployment of DAC faces formidable challenges, notably high capital and operational costs (currently USD 300–USD 1000/tCO_2_), substantial energy demands (1500–2400 kWh/tCO_2_), water interference, scalability hurdles, and sorbent degradation. Furthermore, this review comprehensively examines the burgeoning global DAC market, its diverse applications, and the critical socio-economic barriers to adoption, particularly in developing countries. A comparative analysis of DAC within the broader carbon removal landscape (e.g., CCS, BECCS, afforestation) is also provided, alongside an address to the essential, often overlooked, environmental considerations for the sustainable production, regeneration, and disposal of spent nanomaterials, including insights from Life Cycle Assessments. The nuanced techno-economic landscape has been thoroughly summarized, highlighting that commercial viability is a multi-faceted challenge involving material performance, synthesis cost, regeneration energy, scalability, and long-term stability. It has been reiterated that no single ‘best’ material exists, but rather a portfolio of technologies will be necessary, with the ultimate success dependent on system-level integration and the availability of low-carbon energy. The review paper contributes to a holistic understanding of cutting-edge DAC technologies, bridging material science innovations with real-world implementation challenges and opportunities, thereby identifying critical knowledge gaps and pathways toward a net-zero carbon future.

## 1. Introduction

### 1.1. The Growing Imperative for Carbon Removal

The escalating atmospheric concentration of carbon dioxide (CO_2_), primarily attributed to anthropogenic activities encompassing fossil fuel combustion, deforestation, and industrial processes, constitutes a principal driver of contemporary climate change [1]. This increase in CO_2_ levels precipitates profound perturbations within Earth’s delicate ecosystems, manifesting as global temperature increases, altered meteorological patterns, ocean acidification, and biodiversity disruptions [2,3]. To mitigate these adverse effects and avert catastrophic warming scenarios, concerted global efforts are underway to cut greenhouse gas emissions and explore innovative technologies for carbon removal (CRT) [4,5,6]. It is not a case that the Intergovernmental Panel on Climate Change (IPCC) has emphasized the critical need for CRT to constrain global warming to 1.5 °C above pre-industrial levels [7]. Achieving this objective necessitates not only the curtailment of current emissions but also the active extraction of historical emissions from the atmosphere [8]. Among the multitude of CRT, direct air capture (DAC) has garnered significant attention as a promising approach for sequestering CO_2_ directly from the ambient air [9,10]. Unlike point-source carbon capture, DAC exhibits spatial flexibility, enabling deployment at diverse locations to address diffuse CO_2_ emissions [11,12]. DAC, along with other carbon removal approaches like afforestation and reforestation, offers a potential pathway to achieve net-negative emissions and restore equilibrium to the global carbon cycle [13]. The urgency of carbon removal is underscored by the recognition that certain sectors, including aviation and heavy industry, may prove recalcitrant to complete decarbonization in the near term, even with stringent emissions reduction targets [14]. DAC provides a critical mechanism to offset these residual emissions and address the cumulative impact of past emissions on atmospheric CO_2_ concentrations [15].

**Figure 1:**
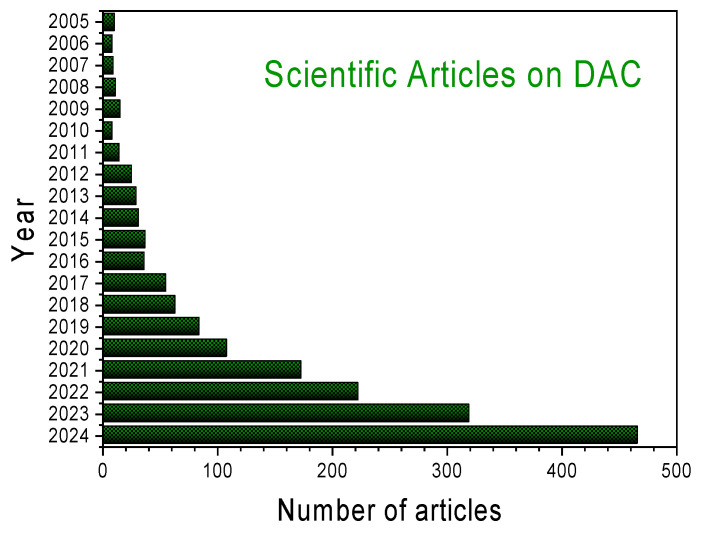
Number of published articles over time with “direct air capture” in the title from ScienceDirect database (March 2025).

The technology typically involves using a sorbent material, either solid or liquid, to selectively capture CO_2_ from the air [16]. The captured CO_2_ is then released in a concentrated stream for various applications, including sequestration in geological formations or utilization in producing valuable products like synthetic fuels and building materials [17,18]. Reflecting this potential, research interest in DAC processes has experienced exponential growth over the past 5–6 years, as evidenced in Figure 1.

### 1.2. DAC Technologies: A Spectrum of Approaches

DAC technologies are broadly categorized as solid DAC (S-DAC) and liquid DAC (L-DAC) [19]. Systems employ solid adsorbents, characterized by high surface areas and/or tailored surface functionalities, to capture CO_2_ molecules. These materials typically operate under ambient to low pressure (vacuum) conditions and are regenerated at moderate temperatures (80–120 °C) [20], a subject to be explored in detail in subsequent sections. Conversely, L-DAC systems utilize aqueous basic solutions for CO_2_ absorption, requiring higher regeneration temperatures (300–900 °C) for CO_2_ release [21]. Within these two broad categories, a diverse range of sorbent materials and process configurations are being explored, each exhibiting unique advantages and disadvantages [22]. The selection of sorbent material and process design is contingent upon factors such as desired CO_2_ capture capacity, selectivity, energy consumption, cost-effectiveness, and environmental impact [23].

As for the liquid sorbent, aqueous monoethanolamine (MEA, 30 wt%) remains the benchmark for point-sources CO_2_ capture. Yet, its low absorption rate and insufficient removal under ultra-diluted CO_2_ concentration (i.e., 400 ppm) [11], high volatility, susceptibility to oxidative degradation [24], environmental concerns, and high regeneration energy requirements [25] make it unsuitable for DAC application. Alkali hydroxide solutions have progressively emerged as viable alternatives for CO_2_ scrubbing [26,27], leveraging the rapid formation of bicarbonate and carbonate species through the reaction of OH^−^ ions with CO_2_. This approach combines rapid absorption kinetics and accessibility with high oxidative stability, low volatility and low toxicity [28]. Lithium hydroxide (LiOH), sodium hydroxide (NaOH), and potassium hydroxide (KOH) have already demonstrated potential for large scale operations (0.5–1 MtCO_2_/year [29]), although the high regeneration temperatures (≈900 °C) remain a primary challenge.

Recent studies have explored environmentally benign alkanolamines, including Pyrrolizidines [30], OXDA, MXDA, and PXDA [31], which exhibit adsorption capacities and rates comparable to NaOH under DAC conditions, potentially offering lower energy penalties during regeneration [32]. Amino acid-based [33] and guanidine-based systems [34] represent the latest advancements in liquid sorbents, demonstrating regeneration temperatures within the 80–120 °C.

Despite these advancements, the high regeneration energy requirements of liquid sorbents pose a significant barrier compared to solid sorbents. Consequently, solid sorbent-based DAC is considered more economically attractive due to its ability to utilize waste heat from industrial processes for regeneration, thereby reducing operational costs.

### 1.3. The Crucial Role of Solid Sorbent Materials

**Figure 2:**
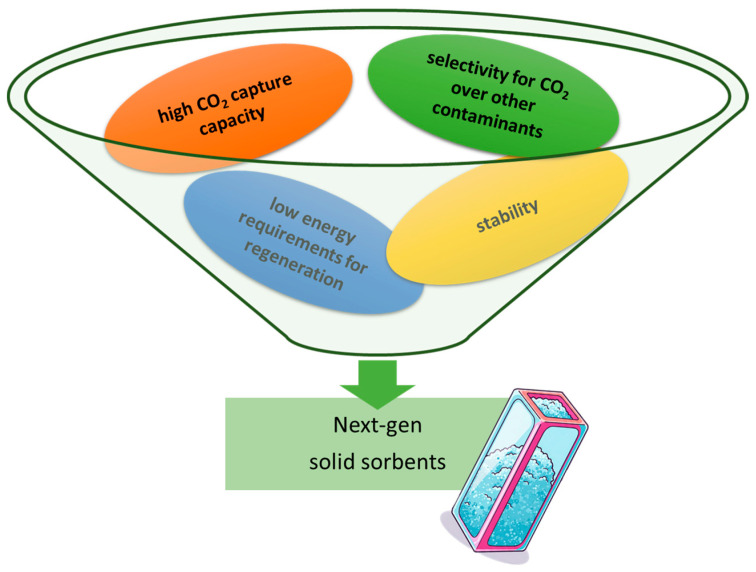
Main features of next-gen solid sorbents.

The development of efficient and cost-effective DAC technologies hinges on the design and optimization of solid sorbents with specific properties (Figure 2) [35]. These materials must exhibit high CO_2_ capture capacity, selectivity for CO_2_ over other gases in the air, stability under various operating conditions, and low energy requirements for regeneration [36]. The capture capacity of a sorbent material determines the amount of CO_2_ captured per unit mass or volume, influencing the size and cost of the DAC equipment [37]. Selective adsorption of CO_2_ over other atmospheric constituents, such as nitrogen and oxygen, is essential to minimize the capture of extraneous gases, which can reduce the purity of the captured CO_2_ stream and increase the energy required for regeneration [38]. Stability ensures that the sorbent material can withstand repeated adsorption–desorption cycles without significant degradation, ensuring consistent performance and longevity [18]. Regeneration energy is a significant contributor to the overall energy consumption and cost of DAC. Minimizing this energy input is essential for economic viability and environmental sustainability of the technology [39].

This Review focuses on the advances in DAC technology, specifically highlighting the use of sorbents for CO_2_ extraction from ambient air. DAC represents a crucial “negative carbon” technology with the potential to mitigate the escalating atmospheric CO_2_ concentrations responsible for climate change. We present a comprehensive overview of various sorbents and processes employed in DAC, with a particular emphasis on Moisture Swing Adsorption (MSA) in Section 3. Additionally, we provide a concise overview of alternative DAC technologies and conclude with a critical analysis and discussion of future perspectives for DAC development and deployment.

## 2. Advanced Sorbent Materials for DAC: Solid and Solid-Supported Systems

Solid sorbents constitute a pivotal class of materials in the realm of DAC technologies, offering inherent advantages such as high surface area, tunable pore structures, and the potential for selective CO_2_ adsorption [40]. These materials play a crucial role in capturing CO_2_ directly from the ambient air, providing a promising avenue to mitigate escalating atmospheric CO_2_ concentrations and addressing climate change. While the primary focus of this section is on traditional solid sorbents, a critical evaluation of advanced hybrid systems and solid-supported materials that incorporate components not strictly classified as bulk solids (e.g., ionic liquids) are also provided. These materials are included due to their integral role in developing next-generation DAC sorbent technologies, often leveraging nanostructured components or principles of nanoscale engineering to enhance efficiency and selectivity.

The pursuit of the ideal solid sorbent for DAC applications, characterized by the extremely dilute CO_2_ concentration (approximately 400 ppm), has been a primary focus of research. This pursuit is driven by the need for materials that exhibit high CO_2_ capture capacity, exceptional selectivity over other atmospheric gases (like N_2_, CH_4_ and H_2_), robust stability under DAC conditions, and low energy requirements for regeneration [41].

**Figure 3:**
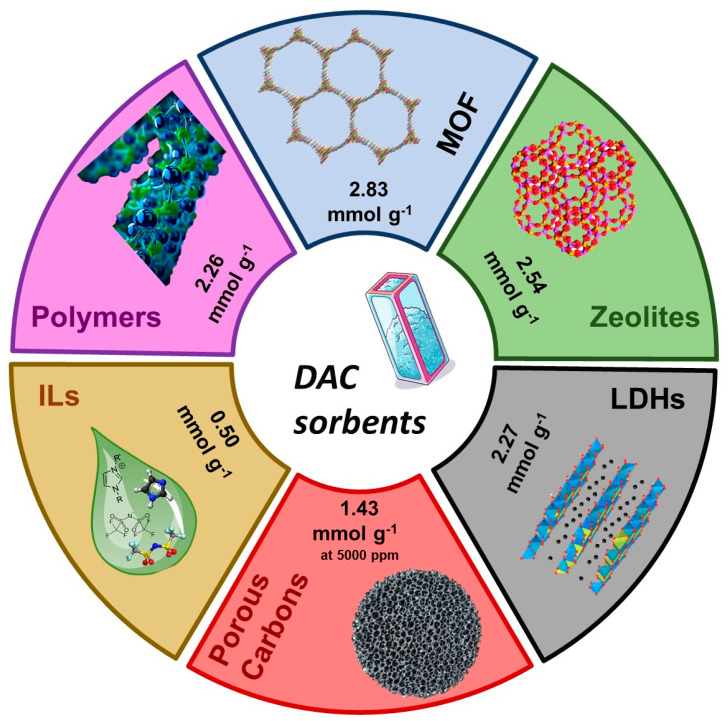
Peak performance of state-of-the-art classes of sorbents for DAC. Each data point represents the highest reported CO_2_ uptake (mmol/g) at 400 ppm CO_2_ concentration, with corresponding citations provided. For enhanced clarity and reproducibility, future iterations of this figure will include detailed annotations specifying the exact temperature (°C), humidity (e.g., dry, 50% RH), and pressure (e.g., ambient, vacuum) at which each peak performance was achieved, alongside the existing direct citations to the original experimental studies for each data point. Peak performance were taken from: MOF [39]; Zeolites [54]; LDHs [72]; Porous Carbons [67]; ILs [78]; and Amine-functionalized Polymers [62].

This review specifically focuses on the most promising and extensively researched classes of nanostructured materials for DAC, including Metal–Organic Frameworks (MOFs), Covalent Organic Frameworks (COFs), Zeolites, Amine-functionalized Polymers, Carbon-Based Materials, Layered Double Hydroxides (LDHs), and Ionic Liquids in solid-supported and hybrid forms, to provide a concise and scientifically reasonable scope of discussion. Beyond describing their individual properties and recent advancements, we will critically compare their performance characteristics—specifically CO_2_ uptake capacity, regeneration energy, scalability, and stability—in the context of DAC applications, highlighting their respective advantages and limitations. Where data from higher CO_2_ concentrations is presented, it is to illustrate potential or comparative performance, but the primary focus remains on their relevance and challenges for direct air capture. Table 1 summarizes the key attributes of these materials while Figure 3 provides a summary on the state-of-the-art materials for DAC.

**Table 1:** CO_2_ capacity, selectivity and key features for various solid sorbents.

Nanomaterial	CO_2_ Capacity (mmol g^−1^)	Selectivity	Key Advantages	Challenges	Regeneration Energy (GJ/tCO_2_)	Regeneration Energy (GJ/tCO_2_)	Regeneration Energy (GJ/tCO_2_)
MOFs	2.0–4.0 [42,43,44]	Very High	Ultra-high surface area, tunable chemistry, high CO_2_ uptake at low pressure, low regeneration temp (70–120 °C) [45]	Stability issues (e.g., moisture sensitivity), high production cost	0.5–18.75 (Solid DAC range) [46]; lowest: 5.76 [45]	35–350 (operational) [45]; 60–190 (specific) [47]	Sensitive to humidity, can degrade; some amine-appended MOFs show ~1.7 mmol/g at 50% RH, some water-enhanced uptake.
COF	0.9–2.1 [48]	Very High	Exceptional chemical and thermal stability, tunable porosity	Scalability and cost-effective synthesis remain significant hurdles	0.5–18.75 (Solid DAC range) [46]	250–600 (current DAC) [49]	Good performance under humid conditions (e.g., 2.05 mmol/g at 50% RH). Exceptional thermal stability.
Zeolites	1–2.8 [50,51,52,53,54,55]	High	High thermal stability, tunable pore size, excellent selectivity for CO_2_	Sensitive to humidity, limited capacity at low CO_2_ concentrations; high desorption temp [56]	0.5–18.75 (Solid DAC range) [46]	300–4000 (general DAC) [57]; 202 (specific SBA-15) [47]	Sensitive to humidity, water reduces recovery/productivity, creates “cold spots”; CO_2_ adsorption efficient at lower temps (30–50 °C) [58].
Polymer-Based	0.5–2.6 [59,60,61,62,63]	Very High	Low cost, scalable, good CO_2_ affinity; diminished degradation under humid conditions	Limited thermal stability, degradation over multiple cycles; substantial regeneration energy	0.5–18.75 (Solid DAC range) [46]; mild regeneration (50–120 °C) [64]	100–590 (NOAK solid) [65]	Maintains performance under humid conditions; humidity can enhance uptake (20–40% RH optimal); capacity decreases at cold temps (−20 °C) [66].
Carbon-based	0.1–0.5 [67]	Moderate	Low cost, high surface area, good thermal stability	Low selectivity for CO_2_ in presence of other gases; weak physical interaction with CO_2_ at low concentrations	0.5–18.75 (Solid DAC range) [46]; low-grade heat (~100 °C) [68]	100–300 (projected) 19; 94–232 (NETL generic) [69]	Weak interaction with CO_2_ under dilute conditions, less effective in humid air (implied). Good thermal stability.
LDHs	0.5–3.0 [70,71,72,73]	Moderate	Low cost, good thermal stability, reversible CO_2_ adsorption; energy-efficient regeneration	Limited capacity/selectivity vs. MOFs/zeolites; long-term stability under fluctuating humidity/temp	0.5–18.75 (Solid DAC range) [46]; regeneration energy penalty is largest cost [74]	400–1000 (near-term DAC) [75]	Amine-modified show impressive capacity under humid conditions (e.g., 3.2 mmol/g at −20 °C, 70% RH) [76]; capacity can decrease at cold temps (~40% at −20 °C) [76].
Ionic Liquids	0.1–0.5 [77,78]	Moderate	Negligible vapor pressure, high chemical/thermal stability, tunable chemistry, low corrosivity	High production cost, limited capacity/selectivity vs. other sorbents; high viscosity	0.5–18.75 (Solid DAC range) [46]; 70–120 °C regeneration [79]; 50% energy reduction for CO_2_ BOLs [80]	500–1000 (current DAC) [75]	Tunable chemistry for optimization; volatility of IL-glycol mixtures assessed at 25 and 55 °C [79].

### 2.1. Metal–Organic Frameworks (MOFs): Structure, Functionalization, and CO_2_ Capture

MOFs are a class of highly porous materials constructed from metal ions or clusters coordinated with organic ligands. This arrangement creates a three-dimensional framework with exceptionally high surface areas and tunable pore sizes, making MOFs attractive candidates for CO_2_ adsorption [81]. Effective capture of CO_2_ from ambient air necessitates sorbents with high CO_2_ affinity, exceptional selectivity over other atmospheric gases, significant adsorption capacity, and robust stability. MOFs can fulfill these criteria by combining high surface area and porosity with customizable pore dimensions and functionalities, often acting as effective physisorbents due to strong van der Waals interactions and confined pore environments. Moreover, MOFs exhibit inherent selectivity for CO_2_ and relatively low regeneration temperatures. Typical regeneration temperatures for MOF-based technologies range between 80 °C and 120 °C, a significant reduction compared to solvent-based systems (e.g., ~900 °C for liquid DAC). This lower temperature requirement can lead to regeneration energy consumption as low as 1.6 kWh/kg-CO_2_, equating to an operational cost of USD 35–350/ton-CO_2_, depending on the energy source [45]. To increase their CO_2_ affinity and selectivity, particularly under dilute conditions, one of the best strategies relies on the incorporation of specific functional groups, such as amines, which enables efficient chemisorption of CO_2_. For example, IRMOF-74-III-(CH_2_NH_2_)_2_, an alkylamine-functionalized MOF, demonstrates a CO_2_ adsorption capacity of 3.2 mmol/g at 25 °C under pure CO_2_ conditions [82]. CALF-20 MOF, another promising MOF, has shown excellent performance in pilot-scale carbon capture plants, combining high CO_2_ uptake at 0.15 bar (3.6 mmol/g) with high selectivity, and stability [83]. A Zn-based MOF with terminal zinc hydride sites has demonstrated CO_2_ capture ability at temperatures exceeding 200 °C, making it suitable for capturing CO_2_ from industrial exhaust streams [84]. NOTT-101/OEt, with a working capacity of 3.8 mol/kg under pure CO_2_, has outperformed several well-known MOFs, such as Mg-MOF-74 and Ni-4PyC [85]. Despite these promising results under pure CO_2_ conditions, direct air capture performance was rarely tested with MOF.

Crucially for direct air capture, Boone et al. constructed a library of core–shell MOFs based on UiO-66 and UiO-67, demonstrating excellent DAC performance and high selectivity of CO_2_ over N_2_ [42]. Lin et al. recently reported an amine-appended MOF (mmen-appended Mg_2_(dobpdc) with (en = ethylenediamine and dobpdc = 4,4′-dioxido biphenyl-3,3′-dicarboxylate) exhibiting a CO_2_ uptake of 2.83 mmol g^−1^ at 0.39 mbar of dry CO_2_ [39] which decreased till ∼1.7 mmol g^−1^ in the presence of 50% relative humidity [43]. This performance currently represents the state-of-the-art DAC using MOF. However, other studies on amine-functionalized MOFs demonstrate stability up to 50% relative humidity when adsorption temperature varies from 25 to 40 °C [86]. Chen et al. developed two series of amine-functionalized zirconium (Zr) MOF-808 variants with improved CO_2_ capture performance, especially under humid conditions [44]. The l-lysine- and tris(3-aminopropyl)amine-functionalized variants (MOF-808-Lys and MOF-808-TAPA, respectively) achieved CO_2_ uptakes of 0.87 and 1.20 mmol/g at 400 ppm and 25 °C under 50% RH conditions. While MOFs, particularly amine-functionalized variants, demonstrate some of the highest CO_2_ uptake capacities under DAC-relevant conditions among solid sorbents, their widespread implementation faces significant challenges. Compared to more thermally stable materials like zeolites or porous carbons, many MOFs exhibit higher sensitivity to water vapor and can suffer from structural degradation, which impacts their long-term stability and regeneration efficiency. Furthermore, their complex synthesis and high production costs present a considerable hurdle for scalability, making them generally more expensive per unit of captured CO_2_ than polymer-based or carbonaceous sorbents. Current costs for MOF-based DAC systems are estimated to be in the range of USD 60–190 per ton CO_2_, though overall DAC costs can range from USD 300 to USD 1000 per tonne of CO_2_, with a target of USD 100/tCO_2_ for widespread adoption [47].

### 2.2. Covalent Organic Frameworks (COFs)

Covalent Organic Frameworks (COFs) represent another class of highly porous crystalline materials, distinct from MOFs, constructed from organic building blocks linked by strong covalent bonds. Their well-defined, tunable pore structures, high surface areas, and exceptional chemical and thermal stability make them promising candidates for gas adsorption, including direct air capture of CO_2_. Despite the limited literature on this class of materials, a novel covalent organic framework (COF-999) recently demonstrates CO_2_ capture performance of 0.96 mmol g^−1^ under dry conditions and 2.05 mmol g^−1^ under 50% relative humidity, both from 400 ppm CO_2_. This sorbent also exhibited exceptional stability, withstanding 100 cycles with no loss of capacity [48]. COFs, while still a nascent field for DAC compared to MOFs or zeolites, show immense promise due to their exceptional chemical and thermal stability, often surpassing that of many MOFs. Their tunable porosity allows for high CO_2_ uptake, and recent studies demonstrate good performance even under humid conditions, addressing a common challenge faced by other porous materials. However, scalability and cost-effective synthesis remain significant hurdles, similar to those encountered with MOFs, limiting their immediate widespread application for large-scale DAC. While specific regeneration energy and cost data for COFs are limited in the literature, they are expected to fall within the broader range for solid sorbents, which typically require 0.5–18.75 GJ/t-CO_2_ for regeneration and current costs ranging from USD 250 to USD 600 per tonne of CO_2_. The industry targets a cost of USD 100/tCO_2_, though this remains an aspirational goal for most technologies [46]. Clearly, COFs represent a nascent field within DAC research. This relative newness means that comprehensive techno-economic data, particularly concerning regeneration energy and long-term operational costs, are not yet as abundant as for more established sorbent classes like MOFs or zeolites. The absence of such specific data poses a challenge for a thorough assessment of their commercial viability. For COFs to transition from promising laboratory materials to scalable DAC solutions, future research must prioritize not only further optimization of their synthesis and CO_2_ capture performance but also detailed techno-economic analyses. These analyses are crucial for attracting the necessary investment and facilitating their large-scale deployment.

### 2.3. Zeolites: Structure, Functionalization, and CO_2_ Capture

Zeolites, a class of crystalline aluminosilicates, exhibit well-defined nanoporous architectures that confer selectivity for CO_2_ based on their unique pore dimensions and topologies. Their inherent thermal and chemical stability, coupled with their cost-effectiveness and abundance, position zeolites as compelling alternatives to MOFs for large-scale carbon dioxide DAC applications. These materials primarily act as physisorbents, adsorbing CO_2_ through weak physical interactions within their porous networks. However, precise modulation of zeolite chemistry is often requisite to optimize DAC performance. Notably, 13X Zeolite, characterized by high microporosity and uniform pore size, has been extensively investigated for CO_2_ adsorption and separation [87]. Yet, introduction of Fe atoms into the 13X framework has been demonstrated to effectively constrict the micropore channels, thereby enhancing CO_2_ adsorption under simulated DAC conditions. For instance, Xiang et al. successfully synthesized an iron-containing 13X zeolite (namely, Fe@13X) that exhibited a CO_2_ capacity of 0.64 mmol/g under simulated air conditions [50]. Furthermore, Fu and Davis developed a MOR-X type zeolite, derived from a commercial zeolite, that displayed promising DAC performance, achieving a CO_2_ capacity of 1.15 mmol/g at 30 °C and 400 ppm CO_2_, alongside high selectivity, rapid adsorption kinetics, low isosteric heat of adsorption, and robust stability [51]. Lithium-exchanged low-silica zeolite X demonstrated a CO_2_ adsorption capacity of 1.34 mmol/g at 298 K and 400 ppm CO_2_ [52] while the lanthanum-exchanged NaX (LaNaX) exhibited improved CO_2_/N_2_ selectivity and excellent cycling stability [53]. Recently, Wilson et al. reported a Na-X zeolite with an exceptional CO_2_ adsorption capacity of 2.54 mmol/g in ambient air under cold climate conditions [54]. A prominent trend in zeolite-based sorbent development involves amine modification, achieved through chemical or physical grafting of amine functionalities onto the zeolite surface to enhance the CO_2_-philicity, capture efficiency and selectivity through chemisorptive interactions. Kumar and co-workers screened several amine-impregnated FAU zeolites, revealing that PEI/FAU achieved CO_2_ capture of 1.54 mmol g^−1^ under humid conditions at 400 ppm CO_2_ with sustained cycling stability over ten cycles [55]. Despite their compelling attributes such as high thermal and chemical stability and cost-effectiveness, zeolites generally exhibit lower peak capture capacities compared to the most advanced MOFs or highly functionalized polymers. Zeolites are typically regenerated by heat, with temperatures often around 100 °C for amine-impregnated variants. Regeneration energy requirements for zeolite-based systems are generally within the 2–18 GJ/ton (0.6–5 MWh/ton) range for current technologies, with some studies indicating total regeneration energy for specific zeolites around 7 GJ/tCO_2_. Costs for solid adsorption-based DAC systems, which include zeolites, are projected to be in the range of USD 88–228 per ton CO_2_ within the next decade, although current costs are higher, typically USD 300–4000 per ton CO_2_ [68]. While their regeneration energy requirements are often moderate, their inherent hydrophilicity can lead to co-adsorption of water, increasing the energy penalty for CO_2_ release. Competitive adsorption of water vapor at the active sites presents a significant challenge for DAC applications, potentially limiting the overall CO_2_ capture efficiency and, consequently, the viability of zeolites for atmospheric CO_2_ sequestration. Studies confirm that water vapor significantly impacts CO_2_ adsorption capacity, with recovery and productivity dropping by up to 30% in the presence of water. While some amine-impregnated FAU zeolites have shown sustained cycling stability under humid conditions, the inherent hydrophilicity of many zeolites can lead to co-adsorption of water, increasing the energy penalty for CO_2_ release. Effective CO_2_ capture on zeolites is often most efficient at lower temperatures (typically around 30–50 °C), and their performance can be evaluated across a wide range of temperatures, from −30 to 50 °C for real-world applications [88].

### 2.4. Amine-Functionalized Polymers

Amine-functionalized polymers (APs), a relatively new class of CO_2_ sorbents, have recently attracted a considerable amount of research attention because of their high CO_2_ uptake capacity and selectivity coupled with tuneable properties and scalable synthesis [89]. These materials are typically synthesized by incorporating amine functionalities into the polymer matrix, facilitating chemisorption of CO_2_ even at low partial pressure. Employing APs in CO_2_ capture offers advantages such as reduced amine leaching compared to traditional liquid amine scrubbing and diminished (or negligible) performance degradation under humid conditions [90,91]. Despite their potential, the literature on APs specifically for DAC remains relatively limited. Alivand et al. [59] demonstrated a CO_2_ uptake of 2.65 mmol g^−1^ by impregnating a meso-macroporous melamine formaldehyde (MF) scaffold with a high loading of tetraethylenepentamine (TEPA, 71 wt%) and under ultra-diluted CO_2_ conditions. Similarly, Samaddoost et al. [60] developed a TEPA-functionalized A400 resin, achieving a CO_2_ adsorption capacity of 1.32 mmol g^−1^ under 390 ppm CO_2_, which further increased till 1.91 mmol g^−1^ upon incorporation of steartrimonium bromide as a surfactant. Sekizkardes et al. explored polymers of intrinsic microporosity (PIMs) featuring amidoxime and amine functionalities. These sorbents exhibited high CO_2_ uptake and processability into various forms, such as fibers and sheets. The PIM-1-AO-TAEA sorbent demonstrated the highest CO_2_ uptake capacity among all PIM-based sorbents to date, achieving 0.8 mmol g^−1^ at 400 ppm CO_2_ [61]. Functionalization of commercial HP20 resin with polyetherimide (PEI) as an amine carrier yielded an optimized CO_2_ capacity of 2.26 mmol g^−1^ at 400 ppm CO_2_ [62]; however, subsequent modification of PEI-HP20 surface with hydrophobic groups reduced the capture ability to 1.6 mmol g^−1^ in ambient air [63]. Amine-functionalized polymers offer distinct advantages for DAC, particularly their lower production cost and greater scalability compared to MOFs or ionic liquids. Their ability to maintain performance under humid conditions also sets them apart from many physisorbent materials like bare zeolites. The regeneration of amine-functionalized polymers typically occurs at mild temperatures (50–120 °C), requiring energy inputs that generally fall within the 0.5–18.75 GJ/t-CO_2_ range for solid sorbents. While specific cost data for polymer-based sorbents vary, the industry is focused on reducing costs to below USD 200/t in the short term for amine-based TVSA methods, with a broader target of USD 100/tCO_2_ [46,64]. However, a key limitation compared to inorganic sorbents (e.g., zeolites, carbons) is their generally lower thermal stability and susceptibility to degradation over multiple adsorption–desorption cycles, which can compromise their long-term performance and increase operational costs. While their CO_2_ uptake can be competitive, but still insufficient to effectively compete with MOFs and zeolites their regeneration energy can still be substantial due to the strong chemical interaction with CO_2_. Despite these limitations, amine-impregnated polymers like PEI- and TEPA-loaded alumina have demonstrated stable and high working capacities across 10 cycles of adsorption–desorption at temperatures ranging from −20 to 25 °C, with desorption at 60 °C. The presence of moisture (e.g., 70% RH) can even improve CO_2_ capacity at both ambient and sub-ambient temperatures for these materials. However, significant degradation (e.g., ~40% decrease in working capacity) is observed after 10 humid DAC cycles at cold temperatures (−20 °C) due to reduced CO_2_ capture kinetics attributed to amine redistribution [66,76].

### 2.5. Carbon-Based Materials

Porous carbonaceous materials present a compelling platform for CO_2_ sorption, leveraging their high specific surface area [92], facile synthesis, tunable pore architecture, cost-effectiveness, and excellent stability [93]. Contemporary research endeavors are directed towards the development of porous carbons with tailored physicochemical properties to enhance CO_2_ capture efficiency and selectivity under DAC conditions [94]. A prominent strategy involves the introduction of heteroatom functionalities, such as nitrogen, oxygen, and sulfur, onto the carbon surface. These functionalities can increase CO_2_ adsorption capacity and selectivity by strengthening interactions between CO_2_ molecules and the carbon matrix. For instance, nitrogen-containing functional groups generate basic sites that facilitate CO_2_ adsorption through acid-base interactions [95]. An alternative approach focuses on the synthesis of hierarchical porous carbon materials, characterized by interconnected micro-, meso-, and macropores. This hierarchical architecture promotes efficient CO_2_ diffusion and mass transfer, thereby improving capture kinetics [96]. Researchers are also investigating diverse activation methodologies, including hard templating, soft templating, and chemical activation, to precisely control the pore size distribution and specific surface area of the carbon materials. Zhang et al. recently reported a KOH-activated bamboo biochar with capture capacity of 3.49 mmol g^−1^ at 25 °C from 1 bar of CO_2_ [97]. While impressive, this performance is under high CO_2_ concentrations and does not directly reflect DAC conditions. Despite these advancements, challenges persist in optimizing the performance and scaling up the production of porous carbon materials for industrial DAC applications. This is due to the weak physical interaction between the non-polar surface of carbon-based sorbents and CO_2_, in particular under ultra-diluted conditions characteristic of ambient air. The PKS-Lignin-P600 sorbent developed by Zhang et al. captured 1.43 mmol CO_2_ per gram of material but at P/P0 = 0.005, namely one order of magnitude higher than the atmospheric concentration of CO_2_ [67]. Consequently, balancing the CO_2_ binding enthalpy with the energy demand for sorbent regeneration remains a critical challenge. Excessive binding strength necessitates high energy input for CO_2_ release, whereas insufficient binding may result in diminished CO_2_ capacity. Researchers are actively addressing this challenge by synthesizing novel porous carbons with tailored surface chemistries and pore structures. In summary, porous carbonaceous materials stand out for their unique material properties and synthetic versatility thus combining low cost, high thermal stability, and inherent scalability, making them attractive from an industrial perspective compared to more complex synthesized sorbents like MOFs. These materials typically require regeneration energy in the range of 0.5–18.75 GJ/t-CO_2_. While current costs for solid sorbent DAC (including carbons) are around USD 500–600/tonne-CO_2_, projected costs aim for USD 100–300/tonne-CO_2_, with a long-term goal of less than USD 100/tonne by 2030 for some technologies [46,64]. However, their primary challenge for DAC lies in the weak physical interaction between their non-polar surface and the extremely dilute CO_2_ in ambient air, leading to generally lower CO_2_ uptake capacities and selectivity compared to amine-functionalized sorbents. This weak interaction means that while regeneration energy might be lower, the overall efficiency for dilute CO_2_ capture can be compromised, as evidenced by a reported capacity of 1.43 mmol CO_2_ per gram at P/P0 = 0.005, an order of magnitude higher than atmospheric CO_2_ concentration. Furthermore, most research on carbonaceous materials has been conducted at ambient temperatures (25 °C) or higher, often under dry conditions, with limited studies exploring their performance at sub-ambient temperatures (−20 °C) or varied humidity [45]. Balancing CO_2_ binding enthalpy with the energy demand for sorbent regeneration remains a critical challenge, as excessive binding strength necessitates high energy input for CO_2_ release, whereas insufficient binding may result in diminished CO_2_ capacity under DAC conditions. Ongoing research is focused on enhancing their performance, reducing their manufacturing costs, and facilitating their scale-up for industrial deployment. By pursuing these research directions, investigators aim to further improve the efficacy and applicability of porous carbon materials in DAC, paving the way for their widespread implementation as a viable carbon capture technology.

### 2.6. Layered Double Hydroxides

Layered Double Hydroxides (LDHs), a class of two-dimensional materials, exhibit significant potential for DAC due to their tunable composition, high specific surface area, and inherent anion exchange capabilities. Their layered structure, composed of positively charged metal hydroxide sheets separated by interlayer anions and water molecules, allows for precise control over material properties, optimizing them for CO_2_ adsorption [98]. Notably, LDHs incorporating alkaline earth metals, such as magnesium and calcium, effectively capture CO_2_ from ambient air under mild conditions [99]. For instance, a Mg-Al LDH with a Mg/Al molar ratio of 3 and CO_3_^−^ as interlayer anions achieved a peak adsorption capacity of 0.319 mmol g^−1^ in pure CO_2_ [100]. The energy-efficient regeneration of CO_2_-saturated LDHs via thermal or vacuum swing adsorption further strengthens their appeal for sustainable DAC. Amine surface modification strategies have proven effective in enhancing CO_2_ uptake capacity and selectivity [70]. Ge et al. synthesized a triamine-grafted (TRI-grafted) Mg-Al-CO_3_ LDHs using scalable fast mixing method, achieving stable capture capacity of 0.545 mmol g^−1^ during 200 simulated DAC cycles [71]. Similarly, surface functionalization with branched poly(ethylenimine) (PEI) allowed the Mg–Al–O MMO to achieve CO_2_ uptake of 2.27 mmol g^−1^ under 400 ppm of CO_2_ [72]. Zhao et al. replaced PEI with tetraethylenepentamine (TEPA) and observed an increase in CO_2_ capture till 3.0 mmol g^−1^ under dry ambient air conditions [73]. LDHs offer advantages such as low cost, good thermal stability, and reversible CO_2_ adsorption, making them potentially more economically viable than some complex organic frameworks. The cost of Mg-Al LDHs is reported to be USD 2–3/kg, with amine-modified variants costing around USD 5/kg. Regeneration energy is a critical factor for LDHs, with the regeneration energy penalty being the largest cost component. While specific energy data for DAC applications are still emerging, LDHs generally fall within the solid sorbent range of 0.5–18.75 GJ/t-CO_2_ for regeneration. Overall DAC costs are currently estimated at USD 400–1000 per metric ton of CO_2_ [76]. However, compared to MOFs or highly functionalized polymers, LDHs generally exhibit more limited CO_2_ capacity and selectivity under DAC conditions. Their long-term stability under fluctuating atmospheric conditions, particularly variations in humidity and temperature, remains a critical challenge that needs to be addressed for practical DAC implementation. Despite this, amine-impregnated LDHs (e.g., MgxAl-CO_3_ LDHs and MgxAl-O MMOs) have demonstrated impressive adsorption capacities under humid conditions (70% RH) at both −20 °C (3.2 mmol/g) and 25 °C (2.0 mmol/g). These materials also show promising regenerability during 10 humid DAC cycles at 25 °C with a <10% decrease in working capacity. However, a notable decrease in working capacity (∼40%) is observed after 10 humid DAC cycles at cold temperatures (−20 °C) due to reduced CO_2_ capture kinetics attributed to amine redistribution. Addressing this requires scalable synthesis methodologies and optimization of adsorption kinetics and capacity for practical DAC implementation. Future research should prioritize exploring novel LDH compositions, developing advanced surface modification techniques, and integrating LDHs into hybrid DAC systems to achieve efficient and cost-effective atmospheric CO_2_ capture.

### 2.7. Ionic Liquids in Solid-Supported and Hybrid Sorbents

Ionic liquids (ILs), defined as organic salts with melting points below 100 °C, have emerged as promising candidates for DAC, offering unique attributes such as negligible vapor pressure, high chemical and thermal stability, minimal corrosivity, non-flammability, and tunable structures [101]. While not strictly solid sorbents themselves, their relevance in the context of this review on advanced sorbent materials and nanomaterials for DAC stems from their increasing exploration in solid-supported forms and hybrid composites, where they leverage the benefits of a solid matrix while contributing their inherent CO_2_ affinity and selectivity. This integration allows for the development of advanced sorbent systems that combine the ease of handling associated with solid materials with the high CO_2_ uptake and tunable chemistry characteristic of ILs, thus providing clear advantages over conventional amine solvents for efficient and sustainable carbon capture processes [102,103].

Thapaliya et al. synthesized tetracationic and dicationic ILs, achieving a CO_2_ capture capacity of 0.433 mol/kg at 298.15 K and 10 bar with 3OEt-Im [104,105]. Chen et al. reported a pyrene based conjugated polymer with [P4444][p-2-O] ionic liquid, able to capture CO_2_ from ambient air with capability of 47.37 μmol g^−1^ and a selectivity of 98.8% [77]. Addressing the high viscosity of ILs, which impedes mass transfer and increases energy demands, researchers have explored IL-glycol mixtures. Adding glycols like ethylene glycol and propylene glycol to ILs reduces viscosity and enhances CO_2_ sorption, with encapsulated mixtures showing significantly reduced volatility (e.g., 0.0002 mmol h^−1^ at 25 °C for [2-CNpyr]/diethylene glycol capsules). While ILs offer unique benefits like negligible vapor pressure and tunable chemistry, their performance across a wide range of temperatures (e.g., −30 to 50 °C for real-world DAC) needs further investigation to ensure consistent capture efficiency [79]. Adding glycols like ethylene glycol and propylene glycol to ILs, such as 1-ethyl-3-methylimidazolium 2-cyanopyrrolide ([2-CNpyr]) reduces viscosity and enhances CO_2_ sorption [79]. To mitigate glycol evaporation, encapsulation techniques have been employed, with [2-CNpyr]/diethylene glycol capsules showing the lowest volatility at 0.0002 mmol h^−1^ at 25 °C. This approach addresses the potential loss of volatile components and ensures the long-term stability of the IL-glycol mixtures.

Furthermore, IL/metal–organic framework (MOF) composites have been developed, leveraging the high surface area of MOFs and the enhanced selectivity and capacity of ILs. Klemm et al. demonstrated CO_2_ chemisorption and microwave-assisted regeneration, in these composites, achieving selective CO_2_ absorption from synthetic air (0.5 mmol g^−1^ at 30 °C) and rapid desorption within 2–4 min [78]. This study demonstrated the synergistic effects of combining different materials to optimize DAC performance: while MOFs provided a high surface area for CO_2_ adsorption, the ILs enhanced the selectivity and capacity of the composite material. As in the case of carbonaceous sorbent, critical challenge in DAC is balancing CO_2_ binding strength with the regeneration energy. Researchers are addressing this by developing novel ILs with tailored properties and exploring alternative regeneration techniques, such as microwave-assisted regeneration [106]. Functionalized ILs, classified as room-temperature ILs (RTILs), task-specific ILs (TSILs), dual-functionalized ILs (DFILs), and amino-acid ILs (AAILs) [107], each offering unique properties for optimized DAC performance. Amino-functionalized ILs, for example, exhibit high thermostability and capture capacity compared to conventional ILs [106]. Ionic liquids provide unique benefits for DAC, notably their negligible vapor pressure and tunable chemistry, which offer advantages over conventional liquid amine solvents in terms of environmental impact and degradation. While their CO_2_ uptake can be competitive, especially in functionalized or hybrid forms, their high viscosity and relatively high production costs present significant challenges compared to most solid sorbents. Regeneration energy for IL-based systems, particularly when integrated with MOFs and utilizing microwave-assisted regeneration, can achieve rapid desorption within 2–4 min. While specific cost data for ILs in DAC are still developing, the overall cost of DAC systems, including those exploring ILs, currently ranges from USD 500 to 1000 per ton CO_2_, with a long-term target of USD 100/tCO_2_. Efforts are focused on reducing costs through learning-by-doing, with projections for solid and liquid sorbent DAC (which ILs can be part of) ranging from USD 226 to 579 per net ton of CO_2_ at the gigatonne scale [46,75]. The development of solid-supported ILs or IL/MOF composites aims to mitigate these issues by combining the benefits of ILs with the ease of handling and higher surface areas of solid materials, potentially offering a more balanced solution for DAC. Ongoing efforts are directed towards improving IL performance, reducing costs, and scaling up for industrial applications [108]. Future opportunities include controlling the interaction with CO_2_ and its transport at the charged or electrified interface with the ionic-liquid electric double layer, as well as integrating CO_2_ capture and conversion with ILs [109].

## 3. Moisture Swing Adsorption (MSA)

Within the broader landscape of DAC technologies, and building upon the detailed discussion of various solid sorbent materials in Section 2, moisture swing adsorption (MSA) stands out as one of the most promising approaches. Its appeal stems from its potential for low energy consumption and cost-effectiveness [9]. Unlike energy-intensive temperature or pressure swing adsorption (TSA/PSA) methods, MSA leverages ambient humidity for CO_2_ capture and release [110]. This innovative process directly capitalizes on the inherent properties of specific solid sorbents, as highlighted in Section 2, which are designed to exhibit a high affinity for CO_2_ under dry conditions and a significantly reduced affinity under humid conditions [10]. Essentially, MSA involves cycling between dry and humid air streams. Dry air allows CO_2_ to adsorb onto the sorbent surface via weak chemical bonds. Subsequent exposure to humid air triggers CO_2_ release, regenerating the sorbent for repeated use. This moisture-driven adsorption/desorption cycle minimizes energy consumption and waste generation, making MSA an attractive approach for sustainable DAC [111]. The effectiveness of MSA relies on the complex interplay between the sorbent material, CO_2_, and water molecules. The regeneration of adsorbent in moisture swing CO_2_ capture technology utilizes the evaporation free energy of water, which can significantly reduce the problem of high energy consumption associated with traditional temperature swing technology [112].

### 3.1. Mechanisms of Moisture Swing Adsorption

**Figure 4:**
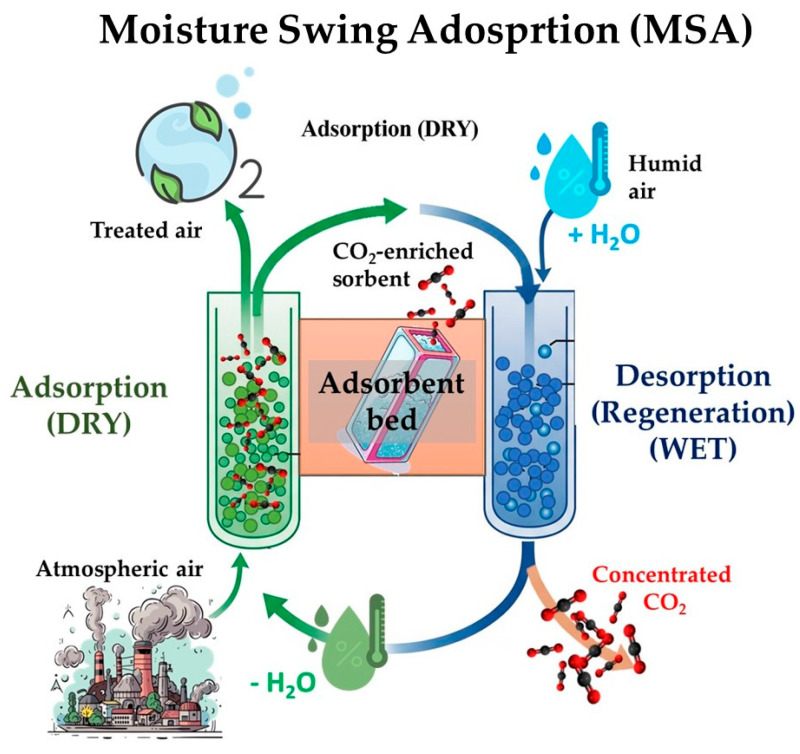
Schematic of mechanisms of moisture swing adsorption method.

The effectiveness of moisture swing adsorption (MSA) relies on the complex interplay between the sorbent material, CO_2_, and water molecules. Several mechanisms govern the moisture-swing behavior of these materials. Some MSA systems relies on the so called “Hydrolysis and Neutralization” mechanism (see Figure 4), according to which water molecules react with anions in the sorbent via hydrolysis, generating hydroxide anions (OH^−^). These serve as active sites for CO_2_ capture through reaction with CO_2_ to form bicarbonates. During regeneration, exposure to high humidity promotes neutralization, where water reacts with bicarbonates and protonated anions, releasing CO_2_ [38]. An alternative mechanism, namely hydration and dehydration, is based on the capability of sorbent materials to undergo structural changes upon hydration and dehydration, impacting their CO_2_ affinity. Some ion-exchange resins, for example, exhibit higher CO_2_ adsorption capacity in their dehydrated state due to increased accessibility of active sites. Conversely, hydration can induce swelling, reducing CO_2_ affinity and promoting its release. Finally, competitive adsorption can occur, where water molecules compete with CO_2_ for adsorption sites, potentially decreasing CO_2_ capture under humid conditions. However, in some cases, water can enhance CO_2_ adsorption by promoting bicarbonate formation or altering the sorbent’s structure.

### 3.2. Sorbent Materials for Moisture Swing Adsorption

The selection of sorbent materials suitable for MSA remains, to date, very limited. Potassium Carbonate solutions, which are liquid-phase systems, are considered the current benchmark for the MSA process. De facto, combining potassium carbonate solutions with guanidine compounds enables rapid and efficient CO_2_ capture from air. In this system, guanidine compounds react with CO_2_-rich potassium carbonate solutions, crystallizing as an insoluble carbonate salt that can be regenerated with minimal energy input [23]. Membrane-based sorbents have emerged as a compelling alternative, demonstrating high efficiency in capturing CO_2_ from ambient air, even at low CO_2_ concentrations. These sorbents typically comprise thin films or porous structures that facilitate rapid CO_2_ adsorption and desorption under varying humidity conditions [111]. Among membrane sorbents, ion-exchange resins (IERs), characterized by quaternary ammonium cations and hydroxide or carbonate counterions, have shown promise [113]. He et al. first demonstrated the effectiveness of a commercial Excellion membrane for MSA, achieving a capture capacity of 0.13 mmol/g in 400 ppm of dry CO_2_ with release under humid conditions [114]. A higher MSA performance of 0.72 mmol g^−1^ was attained using polyHIPE [115]. Recent research has expanded the library of ions for moisture-swing carbon capture, exploring orthosilicate, borate, pyrophosphate, tripolyphosphate, and dibasic phosphate anions in ion-exchange resins [116,117,118,119]. Biery et al. designed a sulfone-based multiblock copolymers containing ammonium functionalities demonstrating their potential for moisture-swing direct air capture of CO_2_. The maximum CO_2_ uptake observed was less than 50 µmol g^−1^, therefore significantly lower than that necessary for commercially viable materials [120]. A novel approach by Nicotera et al. employed a quaternary ammonium-functionalized graphene oxide material, achieving a reversible CO_2_ capture capacity of 3.24 mmol g^−1^ of CO_2_ under simulated MSA conditions [121], representing the highest reported performance to date for MSA sorbents.

### 3.3. Prospects and Challenges of Moisture Swing Adsorption

MSA offers several advantages over traditional DAC technologies. Primarily, it significantly reduces energy consumption compared to TSA/PSA processes by eliminating the need for high temperatures or pressures during sorbent regeneration. This translates to lower operational costs and reduced environmental impact, potentially enhancing the economic viability of large-scale CO_2_ capture [10,18]. Furthermore, MSA’s compatibility with various sorbent materials, including MOFs, zeolites, and amine-functionalized polymers, provides flexibility in material selection and process design. By minimizing waste generation and reducing reliance on fossil fuels, MSA contributes to a more sustainable carbon capture process.

However, challenges remain before MSA reaches maturity [9]. A particularly critical aspect and potential limitation of MSA is its absolute reliance on specific and often high humidity conditions for effective CO_2_ desorption and sorbent regeneration. Unlike other DAC methods that might require dry air or operate best in low-humidity environments, MSA fundamentally leverages the presence of water vapor. This necessitates meticulous control and optimization of the humidity swing, which can add significant complexity to the process design and operation, and potentially lead to energy penalties if humidification or dehumidification of the air stream is required. Desorption kinetics may also be slower compared to thermal regeneration methods, potentially impacting overall DAC efficiency. For instance, typical humidity or temperature swing operations for some materials yield 0.9–1.1 mg of CO_2_ per 1 g per hour, with absorption and desorption times being comparable in humidity swing [122]. Moreover, as a relatively nascent technology, further research and development are crucial to fully explore MSA’s potential and address its limitations, especially concerning the precise management of water within the system.

Despite these challenges, continued research and development in MSA are crucial for advancing its technological maturity and realizing its full potential for DAC applications. Key areas of future focus include exploring new sorbent materials with higher CO_2_ capture capacity, greater selectivity for CO_2_ over other atmospheric components, and enhanced stability under varying humidity conditions, which is essential for improving the performance of MSA systems. This may involve investigating new classes of materials or modifying existing materials to optimize their properties for MSA, A, specifically focusing on materials with tailored water interactions that facilitate efficient CO_2_ release. Additionally, fine-tuning operational parameters, such as the humidity swing, air flow rates, and sorbent bed configurations, can significantly enhance the efficiency and kinetics of CO_2_ capture and release. Advanced modeling and simulation techniques can aid in identifying optimal operating conditions for specific MSA systems, particularly in managing the water cycle efficiently. Furthermore, integrating MSA with other carbon capture and utilization technologies, such as the conversion of CO_2_ into valuable products, can enhance the economic viability and environmental benefits of DAC. This integrated approach can create a closed-loop system where captured CO_2_ is utilized rather than simply stored, further contributing to sustainability goals.

By addressing these challenges and capitalizing on its inherent advantages, MSA has the potential to become a leading technology for DAC, contributing significantly to global efforts in mitigating climate change and achieving a sustainable future.

## 4. Emerging DAC Technologies

While solid sorbents and moisture swing adsorption represent significant advancements in DAC technology, the pursuit of even more efficient and cost-effective CO_2_ removal methods continues. This chapter explores emerging DAC technologies, such as Electro Swing Adsorption (ESA), Passive DAC, carbon dioxide-binding organic liquids (CO_2_ BOLs), and bio-inspired approaches, that hold promise for further improving the efficiency and economic viability of capturing CO_2_ from the atmosphere. To provide a holistic perspective, Table 2 provides a comparative analysis of the various DAC technologies, highlighting their primary mechanisms, advantages, limitations, energy consumption, scalability, and environmental considerations, directly addressing the commercial prospects and trade-offs involved in selecting a DAC technology. It is important to note that while these technologies may not always exclusively rely on nanomaterials, many integrate nanostructured components or leverage principles of nanoscale engineering to significantly enhance their efficiency, selectivity, and overall performance, thereby linking them to the central theme of this review.

### 4.1. Electro Swing Adsorption (ESA)

Electro swing adsorption (ESA) is an emerging DAC technology that leverages electrochemistry for CO_2_ capture and release [38]. ESA systems utilize a solid electrode with high CO_2_ affinity as the sorbent material. Applying a negative charge to the electrode attracts and adsorbs CO_2_ from the air, while a positive charge releases the captured CO_2_ for collection and concentration. This approach offers several advantages. ESA can potentially operate with lower energy consumption than traditional temperature or pressure swing adsorption (TSA/PSA) processes, as it avoids the need for high temperatures or pressures during regeneration. MIT researchers demonstrated a ‘faradaic electro-swing’ that achieved >90% efficiency using only 40–90 kJ per mole of CO_2_, which is roughly 0.9–2.0 GJ per ton, representing up to a 10-fold reduction in energy compared to conventional methods. Other electrochemical systems have shown energy consumption as low as 63 kJ/mol CO_2_. These systems often utilize nanostructured electrode materials, such as porous carbons, metal oxides, and conductive polymers, to maximize surface area for CO_2_ interaction and optimize electrochemical activity [64,123]. The inherent fast kinetics of electrochemical processes facilitate rapid CO_2_ adsorption and desorption, potentially enhancing overall DAC system efficiency. Additionally, the electrode’s CO_2_ selectivity can be tuned by modifying its surface chemistry or applied electrochemical potential. Many of the advanced electrode materials being developed for ESA are nanostructured, including porous carbons, metal oxides, and conductive polymers, designed to maximize surface area for CO_2_ interaction and optimize electrochemical activity. Several companies are actively developing ESA-DAC systems. Verdox is focused on a novel electrochemical cell with a proprietary electrode material, aiming for low energy consumption and high CO_2_ capture capacity [124]. Carbon Atlantics is pursuing a different electrochemical cell design, prioritizing scalability and cost-effectiveness. These efforts highlight the growing interest in ESA as a potentially transformative DAC technology [125]. ESA modules with bipolar electrodes have demonstrated scalability without reducing adsorptive performance, and with improvement in energetic performance, making them attractive for gigaton-scale carbon capture.

### 4.2. Passive DAC

Passive DAC is another innovative approach that accelerates the natural carbon mineralization process, leveraging the reaction between calcium hydroxide and atmospheric CO_2_ to form limestone (calcium carbonate) [112]. In a typical passive DAC system, calcium hydroxide is typically exposed to the atmosphere in a controlled environment, passively absorbing CO_2_ from the air. The resulting calcium carbonate can be processed to release the captured CO_2_ and regenerate the calcium hydroxide for subsequent cycles. This technology offers potential advantages in terms of energy efficiency and cost-effectiveness, as it relies on a spontaneous chemical reaction rather than energy-intensive processes [126]. While the term ‘passive’ implies minimal energy input, the efficiency of passive DAC systems can be significantly improved by utilizing calcium hydroxide or other reactive materials in high surface area, often nanostructured, forms to maximize the contact area with atmospheric CO_2_ and accelerate the carbonation kinetics. This approach fundamentally aims for near-zero operational power consumption for the capture step itself, similar to passive DAC cables that consume no power and reduce heat. While fundamentally a chemical reaction, the efficiency of passive DAC systems can be significantly improved by utilizing calcium hydroxide or other reactive materials in high surface area, often nanostructured, forms to maximize the contact area with atmospheric CO_2_ and accelerate the carbonation kinetics. Passive DAC systems has recently gained the attention of several companies. Carbon Collect, for instance, is focused on a contactor-based system with an emphasis on modularity and scalability [64]. Heirloom Carbon Technologies is developing a system that utilizes renewably powered kilns to accelerate the carbonation of calcium hydroxide, aiming to combine low energy consumption with cost-effectiveness [126]. These developments underscore the growing interest in passive DAC as a potentially sustainable and scalable approach to CO_2_ removal. However, the scalability of such systems for gigatonne CO_2_ removal requires substantial investment and infrastructure development for material production and handling. While the capture step is passive, the regeneration of calcium hydroxide typically involves high-temperature calcination (e.g., 900 °C), which is energy-intensive and constitutes a significant challenge for achieving net-negative emissions if powered by fossil fuels [127].

### 4.3. CO_2_ BOLs

**Figure 5:**
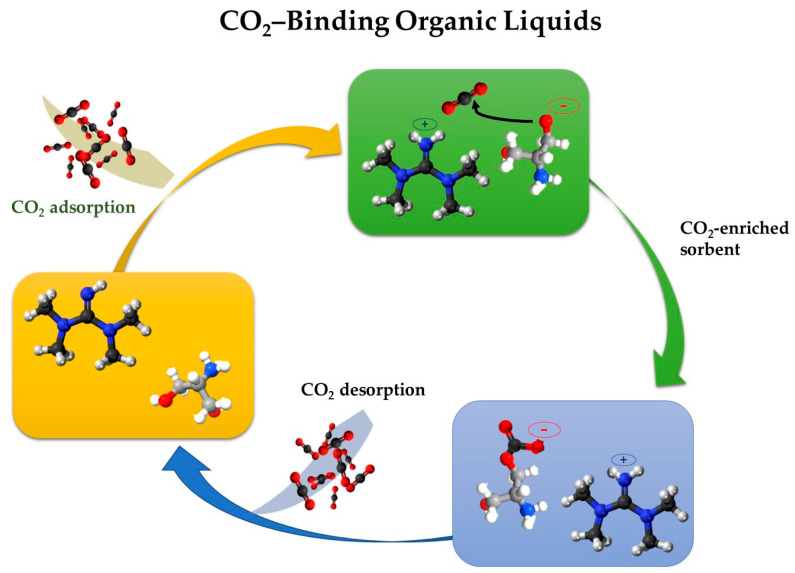
Schematic of mechanisms of carbon dioxide-binding organic liquids (CO_2_ BOLs).

Carbon dioxide-binding organic liquids (CO_2_ BOLs) represent a novel approach to the efficiency of DAC by lowering the enthalpy of sorbent regeneration. CO_2_ BOLs chemically bind and release CO_2_ more energetically and efficiently than aqueous alkanolamine systems. They have high CO_2_ capacities (19% by weight, 147 g CO_2_/L) compared to 30% monoethanolamine solution in water (7% by weight, 108 g CO_2_/L) because they are liquid with or without CO_2_ and do not require any added solvent such as water. The specific heats of organic CO_2_ BOLs are over 50% lower than water, resulting in a 50% reduction in energy to strip out CO_2_ compared to aqueous alkanolamine solutions [80]. These liquids are designed to selectively bind with CO_2_ molecules, forming stable complexes that can be easily separated and regenerated as presented in Figure 5. Typically, CO_2_ BOLs comprise a mixture of an alcohol and a strong organic base, such as 1,8-Diazabicyclo[5.4.0]undec-7-ene (DBU) or 1,1,3,3-Tetramethylguanidine (TMG), coupled with alcohols like hexanol or propanol, which upon CO_2_ uptake form liquid amidinium or guanidinium alkylcarbonate salts [80]. Some CO_2_ BOLs also exist as single-component systems, where the alcohol and basic moieties are combined in one molecule, such as alkanolguanidines and alkanolamidines [128]. A key strategy for deploying CO_2_ BOLs in DAC involves immobilizing them with porous support, often nanostructured materials like mesoporous silica of MOFs [23]. This creates solid-supported CO_2_ BOLs, combining the high CO_2_ capture capacity of liquid sorbents with the ease of handling and regeneration associated with solid sorbents and leveraging the high surface area of the nanomaterial support. However, a significant challenge for CO_2_ BOLs, particularly for large-scale deployment, is managing the sharp increase in viscosity after saturated absorption, which can lead to equipment scaling and pipe blockage. While adding glycols can mitigate this, maintaining long-term stability and preventing volatile component loss remains a concern. Ongoing research is dedicated to developing and optimizing CO_2_ BOLs for DAC applications. The Pacific Northwest National Laboratory (PNNL) is focused on lowering the energy requirements for sorbent regeneration, aiming to improve the economic viability of DAC technologies [129]. Researchers at the UNCAGE-ME Energy Frontier Research Center are exploring the use of CO_2_ BOLs in porous liquids for DAC, with a focus on understanding their fundamental properties and optimizing their performance for CO_2_ capture [129]. These research initiatives highlight the potential of CO_2_ BOLs to contribute to the advancement of DAC technologies.

### 4.4. Comparative Analysis of Direct Air Capture Techniques

The diverse array of DAC technologies, each with distinct Technology Readiness Levels (TRLs), energy requirements (thermal vs. electrical), and operational characteristics, underscores a fundamental truth: a portfolio approach is essential for gigaton-scale DAC deployment. There is no single “best” DAC solution universally applicable to all scenarios. The optimal choice for deployment is highly dependent on a complex interplay of regional factors, including the availability of low-cost, low-carbon energy (whether thermal or electrical), access to water resources, the presence of suitable geological storage sites, and specific local climate conditions (e.g., prevailing humidity and temperature) [49,130,131]. For example, while solid sorbent DAC is currently at a higher TRL (7–9) compared to liquid DAC (4–6) and electrochemical methods (1–4), liquid DAC requires very high regeneration temperatures (300–900 °C), while solid DAC operates at more moderate temperatures (80–120 °C) and can potentially leverage waste heat [132]. Electro Swing Adsorption aims to reduce thermal energy by relying on electrical energy, and Moisture Swing Adsorption leverages ambient humidity for regeneration. The source of energy for a DAC plant significantly impacts its net climate benefit; for instance, using nuclear energy for a solid sorbent DAC plant results in nearly four times lower lifecycle emissions than using natural gas with recapture for a liquid sorbent plant. This implies that merely showcasing a nanomaterial’s high CO_2_ uptake capacity is insufficient; the discussion must extend to how these materials integrate into larger DAC systems and how their specific properties can be leveraged within a broader energy and economic framework to achieve cost reduction [64]. Material science innovations are a necessary but not sufficient condition for cost-effective DAC; parallel advancements in systems engineering and supportive policy are equally vital. Therefore, to achieve the necessary gigaton-scale CO_2_ removal, a diverse portfolio of DAC technologies will likely be indispensable. This necessitates sustained support for research and development across various approaches to ensure flexibility and resilience in addressing the multifaceted challenges of global DAC deployment.

**Table 2:** Comparative analysis of direct air-capture technologies.

DAC Technology	Primary Mechanism	Key Advantages	Key Limitations	Typical Regeneration Energy (GJ/tCO_2_)	Typical Cost (USD/tCO_2_)	Technology Readiness Level (TRL)	Scalability Potential	Water Usage(tons H_2_O/ton CO_2_)	Environmental Considerations
**Solid DAC**	Adsorption (Physisorption/Chemisorption)	Moderate regeneration temp (80–120 °C), waste heat utilization, modular for smaller plants	Slower kinetics, water sensitive, high sorbent capital cost	Thermal: 2–6 [133]; Electrical: 0.3–1.0 [133]	100–590 (NOAK) [65]; 500–600 (current) [134]	7–9 (Commercial Demonstration) [133]	High, scales linearly [65]	1–12 (general DAC)	Net GHG reduction: 640 kg CO_2_-eq/t CO_2_ [135]
**Liquid DAC**	Absorption (Chemical Reaction)	Rapid kinetics, high oxidative stability, large-scale operations (0.5–1 MtCO_2_/yr)	High regeneration temp (300–900 °C), high energy demand, sorbent degradation/loss 7	Thermal: 6–9 [64]; Electrical: 0.2–0.5 [136]	230–355 (current) [65]; 94–232 (projected) [137]	4–6 (Bench Scale/Prototype) [133]	High, economies of scale [65]	More water intensive than S-DAC [46]; net GHG reduction: 560 kg CO_2_-eq/t CO_2_.45	
**Moisture Swing Adsorption (MSA)**	Adsorption (Humidity-driven)	Low energy consumption, cost-effective, no high temp/pressure, minimal waste	Reliance on specific humidity, slower kinetics, limited sorbent selection, nascent technology	Utilizes water evaporation energy (lower than TSA) [138]	Lower operational costs (implied)	Early stages (implied, nascent)	Moderate to High [122]	High (leverages water for regeneration)	More sustainable, reduced reliance on fossil fuels (implied)
**Electro Swing Adsorption (ESA)**	Electrochemical Adsorption/Desorption	Potentially lower energy, fast kinetics, tunable selectivity, renewable electricity compatible	Relies on electrical energy, complex electrode design, lower capacity than amines, uncertainties in cost/supply	0.9–2.0 [47]; as low as 63 kJ/mol [139]	Unclear, but aims for lower costs [131]	1–4 (Lab to Bench) [132]	High, scalable via bipolar electrodes [130]	Not explicitly stated, but generally lower than liquid systems.	Can be driven by renewable electricity, lower environmental footprint [123]
**Passive DAC**	Mineralization (Spontaneous Chemical Reaction)	Energy-efficient (spontaneous reaction), cost-effective, leverages natural processes	Still developing, focus on scalability/cost-effectiveness	Lower energy demand than TVSA [131]	Aims for cost-effectiveness	Early stages (implied)	Companies focused on modularity/scalability	No fresh water needed [131]	Accelerates natural carbon cycle, potentially low environmental footprint
**CO** **_2_** **-binding organic liquids (CO** **_2_** **BOLs)**	Absorption (Chemical Binding/Release)	Lowers regeneration enthalpy, selective CO_2_ binding, negligible vapor pressure, tunable structure	High viscosity, high production costs	50% energy reduction vs. alkanolamines [80]	High production costs	Early stages (implied)	Ongoing efforts to scale up	Not explicitly stated.	Advantages over conventional amine solvents in environmental impact/degradation

## 5. Challenges and Perspectives of DAC

**Figure 6:**
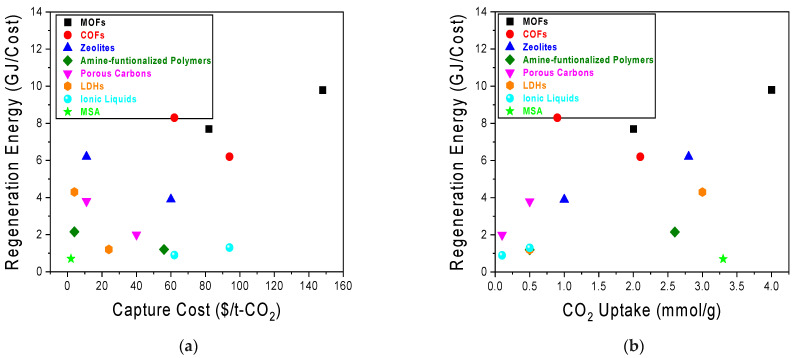
Regeneration energy vs. (**a**) capture cost and (**b**) CO_2_ uptake for various classes of DAC sorbents.

Despite the significant potential of DAC technologies, particularly those employing advanced nanomaterials, several formidable challenges need to be addressed for their widespread deployment and large-scale implementation [140]. It is crucial to understand that there is no single ‘best’ material universally applicable for DAC; the optimal choice depends on specific deployment conditions, energy availability, and economic models. High CO_2_ uptake performance alone does not automatically translate to commercial viability. Commercial viability for DAC involves a complex interplay of high CO_2_ uptake, low regeneration energy, cost-effective and scalable synthesis, robust long-term stability, and water resistance. No single material excels in all aspects, necessitating a holistic techno-economic assessment rather than focusing solely on CO_2_ uptake. The DAC challenges are often exacerbated by the unique properties and requirements of nanomaterials in the demanding DAC environment:

- High cost: DAC remains a relatively expensive technology compared to other carbon capture methods. The cost of capturing CO_2_ from the air is significantly higher than capturing it from point sources due to the low concentration of CO_2_ in the atmosphere [141]. Recent modeling studies assume DAC costs as low as USD 100 to USD 200 per ton of CO_2_ removed, but evidence suggests far higher costs. Currently, DAC and mineralization costs range from ** USD 300 to USD 500 per tonne of CO_2_, and can be as high as USD 1000 in older technologies **. The industry often targets bringing costs down to ** USD 100/tonne of CO_2_ ** for widespread adoption, though current demonstrated costs are closer to ** USD 1000/tonne of CO_2_ **. First-of-a-kind (FOAK) DACCS projects (DAC coupled with storage) are likely to range from ~USD 400 to USD 700/net-tCO_2_ using global average solar photovoltaics (PV) costs, or ~USD 350 to USD 550/net-tCO_2_ with lowest-cost renewables. Significant cost reductions could potentially lead to ~USD 194–USD 230/net-tCO_2_ for 1 MtCO_2_/year scale for nth-of-a-kind (NOAK) DACCS plants. For nanomaterials, this cost is often driven by the complex synthesis procedures, high-purity precursors, and energy-intensive fabrication processes required to achieve desired structural and chemical properties. The sorbent material itself can account for as much as 80% of all DAC costs [75]. For instance, the multi-step synthesis of many MOFs and COFs contributes significantly to their high capital cost per ton of CO_2_ removed, making them generally more expensive than simpler carbon-based or polymer sorbents. While some materials like zeolites and LDHs offer lower production costs, achieving the necessary DAC-specific performance often requires costly functionalization or post-synthetic modification, further impacting the overall economic viability of these nanomaterial-based systems.
- Energy consumption: Some DAC technologies, particularly those based on liquid sorbents, require high energy input for sorbent regeneration. This can offset the environmental benefits of CO_2_ removal if the energy is sourced from fossil fuels [142]. Most proposed processes require an equivalent of at least 1.2 megawatt-hours of electricity for each tonne of CO_2_ removed. Current DAC systems are more specifically reported to consume 1500–2400 kWh per ton of CO_2_. For comparison, the theoretical minimum energy consumption for DAC is 0.5–0.76 GJ per ton of CO_2_ (or approximately 140–210 kWh per ton of CO_2_), highlighting the significant gap between current practice and theoretical limits. Most proposed processes require an equivalent of at least 1.2 megawatt-hours of electricity for each tonne of CO_2_ removed. Overall, the work equivalent regeneration energy demand for solid sorbent DAC systems ranges from 0.5 to 18.75 GJ/t-CO_2_, and for liquid solvent DAC systems, it ranges from 0.62 to 17.28 GJ/t-CO_2_. Energy costs can constitute as much as 50% of long-term liquid DACCS costs. For solid sorbents, regeneration temperatures are typically 80–120 °C, while liquid sorbents require much higher temperatures (300–900 °C) for CO_2_ release. Continuous fan operation and sorbent regeneration are major contributors, consuming nearly 2000–3000 kWh per ton of CO_2_ captured. For comparison, the theoretical minimum energy consumption for DAC is 0.5–0.76 GJ per ton of CO_2_ (or approximately 140–210 kWh per ton of CO_2_), highlighting the significant gap between current practice and theoretical limits. Energy costs can constitute as much as 50% of long-term liquid DACCS costs. While solid sorbents generally require lower regeneration temperatures than liquid systems, the energy demand can still be substantial. Figure 6a provides a quick overview about the “Regeneration Energy (GJ/Cost)” against “Capture Cost (/T_CO_2__)” and “CO_2_ Uptake (mmol/g)” for the various classes of DAC sorbents treated in the review. For chemisorbents (e.g., amine-functionalized MOFs, polymers, or zeolites), the strong binding with CO_2_ necessitates higher energy input for desorption, often requiring thermal swing adsorption (TSA). This strong interaction, while beneficial for capture efficiency, directly translates to a higher energy penalty during regeneration. In contrast, physisorbents (like many pristine carbons or MOFs) might require lower regeneration temperatures due to weaker CO_2_ interactions, but often suffer from lower CO_2_ capacities under dilute conditions. This means larger volumes of air must be processed and more sorbent cycled, indirectly increasing overall energy consumption for a given amount of captured CO_2_. Emerging technologies like Electro Swing Adsorption (ESA) aim to reduce thermal energy requirements, but still rely on electrical energy, and their electrode materials often involve complex nanomaterial designs that require careful optimization for energy efficiency.

- Water sensitivity: The presence of water vapor in ambient air (typically 50–90% RH) poses a critical challenge for many nanomaterials. Between 1 and 50 tons of water are consumed per ton of CO_2_ captured in almost all DAC approaches. Many highly porous nanomaterials, such as pristine MOFs and zeolites, are inherently hydrophilic, leading to competitive adsorption of water over CO_2_. This significantly reduces CO_2_ capture efficiency and increases regeneration energy as co-adsorbed water must also be desorbed. For instance, CO_2_ recovery and productivity for zeolites can drop by 30% in the presence of water. Developing water-resistant sorbents or efficient dehumidification systems is crucial for improving the performance of DAC systems [88]. Many highly porous nanomaterials, such as pristine MOFs and zeolites, are inherently hydrophilic, leading to competitive adsorption of water over CO_2_. This significantly reduces CO_2_ capture efficiency and increases regeneration energy as co-adsorbed water must also be desorbed [143]. Developing water-resistant sorbents or efficient dehumidification systems is crucial for improving the performance of DAC systems [144]. For instance, amine functionalization can improve CO_2_ selectivity in humid conditions for some materials (e.g., amine-modified polymers, MOFs), but the long-term stability of these functional groups in the presence of moisture and oxygen remains a concern for sorbent degradation, particularly for organic components. However, some amine-impregnated LDHs and polymers have demonstrated impressive CO_2_ uptake under humid conditions, with water even enhancing CO_2_ adsorption for certain MOFs up to an optimum humidity. This suggests a more complex, nuanced interaction where water can be both a challenge and, in some cases, a facilitator for CO_2_ capture, depending on the sorbent chemistry and operating conditions [76,145]. Developing nanomaterials with robust hydrophobic surfaces or selective water exclusion mechanisms is paramount.
- Scalability: Scaling up DAC technologies to capture gigatonnes of CO_2_ from the atmosphere requires substantial investment and infrastructure development [146]. Recent modeling studies project DAC deployment on the scale of 5 to 40 gigatonnes of CO_2_ removed per year, but the likelihood of deploying DAC at the gigatonne scale is highly uncertain. To capture 1% of annual global CO_2_ emissions, one incumbent technology would require ~20% of the global silica and ethanolamine markets. Furthermore, ~25% of global energy supplies by 2100 could be required for gigaton-scale DAC. The transition from laboratory-scale synthesis to industrial-scale production often presents significant challenges related to consistency, reproducibility, and cost-effectiveness, especially for complex, highly ordered materials like MOFs and COFs. In the case of nanomaterials, the transition from laboratory-scale synthesis to industrial-scale production often presents significant challenges related to consistency, reproducibility, and cost-effectiveness. While materials like porous carbons and some polymers are more readily scalable due to established manufacturing processes, the complex, multi-step synthesis of highly ordered materials like MOFs and COFs, or the precise functionalization of others, can be difficult to achieve at the required volumes without compromising performance or drastically increasing costs [146]. Furthermore, engineering contactor designs that efficiently utilize the high surface area of nanomaterials while minimizing pressure drop and material attrition at large scales is a critical area of ongoing research. While solid DACCS costs scale more linearly with size and are likely to be the more cost-effective option for smaller plants (<100 ktCO_2_/year), liquid systems are significantly more cost-effective at large scales due to economies of scale. Electrochemical DAC systems, such as ESA, have demonstrated that their modules can be scaled without reducing adsorptive performance, with improved energetic performance [130].
- Sorbent degradation: The long-term stability of sorbent materials under repeated adsorption–desorption cycles, fluctuating temperatures, and the presence of atmospheric contaminants (like oxygen and trace pollutants) is crucial. Amine-functionalized polymers and MOFs can be susceptible to oxidative degradation, amine leaching, or structural collapse, respectively, leading to a reduction in CO_2_ capture capacity and lifespan. This degradation necessitates frequent sorbent replacement, increasing operational costs and environmental footprint. Inorganic sorbents like zeolites and LDHs generally offer higher thermal stability but may still face issues with hydration/dehydration cycles, or poisoning by other atmospheric components over extended operation. Research indicates that when sorbent degradation is included in models, there is a non-linear relationship between CO_2_ captured and sorbent lifetime as the sorbent’s capacity decreases each cycle. This implies a continuous, rather than sudden, drop in performance. Such degradation necessitates frequent sorbent replacement, increasing operational costs and environmental footprint, and can also lead to an increase in the energy carbon footprint over prolonged use due to reduced efficiency. For economic viability, a typical sorbent must survive tens, if not hundreds, of thousands of loading and unloading cycles [147]. Ensuring the robustness of nanomaterial structures and functional groups under real-world DAC conditions is a key research priority.
- Uncertainty in carbon prices: The long-term value of carbon credits for DAC is uncertain, adding risk to the economic viability of DAC projects, irrespective of the material used. This economic uncertainty can deter investment in the development and deployment of novel nanomaterial-based DAC systems.

Overcoming these challenges requires continued research and development in various areas, including in the following areas:

- Developing new sorbent materials: Researchers are actively exploring new materials with higher CO_2_ capture capacity, selectivity, and stability, as well as lower energy requirements for regeneration. This involves designing nanomaterials with enhanced hydrophobicity to resist water interference, more robust frameworks to withstand repeated cycling, and tailored surface chemistries for stronger yet reversible CO_2_ binding at ambient concentrations [148]. Future directions include exploring novel hybrid nanomaterials that combine the strengths of different classes (e.g., MOF-polymer composites, carbon-supported ionic liquids) to overcome individual limitations.
- Optimizing DAC processes: Improving the efficiency of DAC processes, such as air contactor design and sorbent regeneration methods, can reduce energy consumption and costs [9]. This includes developing novel contactor designs that maximize interaction with nanomaterials while minimizing pressure drop and ensuring efficient mass transfer, as well as exploring alternative regeneration methods like electro-swing or moisture-swing adsorption that are particularly well-suited for certain nanomaterial properties.
- Integrating DAC with other technologies: Combining DAC with other carbon capture and utilization technologies, such as CO_2_ conversion to valuable products, can enhance the economic viability of DAC [18]. CO_2_ utilization technologies aim to convert captured CO_2_ into valuable products or directly use it as a chemical feedstock in various industries, including chemicals and fuels, building materials, enhanced oil recovery (EOR), and bio-products. While EOR remains dominant in terms of scalability, maturity, and economic benefits, photocatalytic and electrochemical reduction of CO_2_ along with bio-fixation are gaining attention for their energy intensity and environmental benefits. However, most promising CO_2_ utilization techniques are either technologically immature or limited in scale to deploy globally, and the high cost of CO_2_-based production and the low value of the CO_2_ market remain significant barriers [149]. Nanomaterials can play a dual role here, not only capturing CO_2_ but also potentially acting as catalysts for its conversion into valuable chemicals or fuels.
- Policy support: Government policies and incentives can play a crucial role in accelerating the development and deployment of DAC technologies [1]. Targeted policies that support fundamental research into nanomaterials for DAC, as well as incentives for pilot-scale demonstrations and commercialization, are essential.
- Public perception: Building public trust and acceptance of DAC technologies is crucial for their widespread deployment. Clear communication of the benefits and risks of DAC, as well as engagement with stakeholders, is essential, including transparent discussions about the lifecycle impacts of nanomaterial production and disposal.

By addressing these challenges and capitalizing on the opportunities, DAC can become a viable and scalable solution for removing CO_2_ from the atmosphere and contributing to a sustainable future. Continued innovation in nanomaterial science will be a cornerstone in realizing the full potential of DAC.

### CO_2_ Utilization Pathways

Nanomaterials, beyond their primary role in CO_2_ capture, are increasingly recognized for their potential to act as catalysts in various CO_2_ utilization pathways, facilitating the conversion of captured CO_2_ into valuable chemicals and fuels. CO_2_ utilization technologies aim to convert captured CO_2_ into valuable products or directly use it as a chemical feedstock in various industries, including chemicals and fuels, building materials, enhanced oil recovery (EOR), and bio-products. While EOR remains dominant in terms of scalability, maturity, and economic benefits, photocatalytic and electrochemical reduction of CO_2_ along with bio-fixation are gaining attention for their energy intensity and environmental benefits. However, most promising CO_2_ utilization techniques are either technologically immature or limited in scale to deploy globally, and the high cost of CO_2_-based production and the low value of the CO_2_ market remain significant barriers [135,149].

While the integration of Direct Air Capture (DAC) with CO_2_ utilization pathways is proposed as a means to enhance economic viability, a significant maturity mismatch currently exists. Most promising CO_2_ utilization techniques, such as photocatalytic and electrochemical reduction or bio-fixation, are either technologically immature or limited in scale for global deployment. This immaturity means they are not yet ready for large-scale integration with DAC. Furthermore, the high cost associated with capturing and concentrating CO_2_ via DAC often renders CO_2_-derived products uncompetitive with conventionally produced alternatives, thereby limiting market demand and the overall value of captured CO_2_. To fully realize the economic benefits of DAC, simultaneous and aggressive investment is needed in both DAC technology and the maturation of CO_2_ utilization pathways. Policy interventions, such as robust carbon pricing mechanisms and targeted subsidies for green technologies, are crucial to bridge this economic gap and stimulate market demand for CO_2_-derived products, fostering a more robust circular carbon economy.

## 6. Real Market and Practical Application of DAC

The global market for Direct Air Capture (DAC) is experiencing significant growth, driven by the pressing need to achieve net-zero emissions and combat climate change. The market for DAC devices was valued at USD 25.9 million in 2024 and is anticipated to reach USD 1340 million by 2031, witnessing a compound annual growth rate (CAGR) of 76.8%. Broader market estimates for Direct Air Capture technology indicate a valuation of USD 93.1 million in 2024, projected to reach USD 2046.3 million by 2034, at a CAGR of 40.4% [150]. Another projection estimates the global DAC market size at USD 74.59 million in 2023, expected to reach USD 8936.42 million by 2033, growing at a CAGR of around 61.38%. This accelerated market momentum over the past five years is attributed to pilot projects transitioning into demonstration-scale facilities, meaningful declines in cost trajectories, and the crystallization of strategic partnerships [151].

Key driving factors for this market expansion include ambitious carbon removal mandates from governments and corporate net-zero commitments, which are catalyzing offtake agreements and integrating capture capacity into broader climate strategies. ^1^ Public–private partnerships are becoming increasingly prominent, with regulatory frameworks adapting to support infrastructure development and provide revenue-stabilizing incentives such as carbon credits and tax credits. The emergence of carbon removal marketplaces has also created transparent valuation mechanisms, enabling project developers to secure long-term financing. Furthermore, continuous development and research are making DAC technology more cost-effective and efficient, with advances in sorbent materials and system design lowering energy intensity and making operations more sustainable and scalable [151].

DAC technology finds applications across various end-user industries. The energy industry is anticipated to dominate the market share, utilizing DAC to capture CO_2_ emissions from power plants and other facilities. In transportation, DAC can be used to produce sustainable synthetic fuels or capture emissions from sources like ships and airplanes. The industrial sector can use captured CO_2_ as a feedstock for manufacturing chemicals, materials, and other goods, thereby lowering their overall carbon footprint. In agriculture, DAC systems offer a consistent supply of CO_2_ for greenhouse operations, enhancing crop growth and production. Other applications include construction, waste management, and R&D endeavors focused on carbon sequestration and utilization.

Leading companies and startups are actively developing and deploying DAC solutions. Climeworks AG, a Swiss company, debuted the world’s first commercial DAC machine in Switzerland and has 13 other commercial plants across Europe, capturing CO_2_ for geological storage or use in food, beverages, greenhouses, and renewable fuels. CarbonCapture Inc. (Los Angeles, CA, USA) develops modular DAC machines, while Heirloom Carbon Technologies (Brisbane, CA, USA) focuses on accelerating calcium hydroxide carbonation using renewably powered kilns. Other notable players include Verdox (Woburn, MA, USA), with electrochemical cells; Carbon Atlantics (Dartmouth, Nova Scotia, Canada), prioritizing scalability; Noya (Oakland, CA, USA), developing cost-effective approaches using abundant materials; South Ocean Air (Houston, TX, USA), utilizing cellulose-based sorbents; Aerbon (Brännkyrkagatan, Stockholm, Sweden), with solid sorbents and waste heat; Yama (Paris, France), building modular systems with hybrid electrochemical and low-heat processes; and Fortyfour (Zurich, Switzerland), providing electric DAC systems for greenhouses.

Despite this global momentum, the adaptability of DAC for developing countries with limited resources faces significant challenges [149]. The high cost of DAC technology is a primary restriction, with current costs being two to six times higher than desired for widespread deployment. Capital and energy costs constitute about 95% of the total DAC technology cost. For many developing nations, the substantial upfront investment and the energy-intensive nature of DAC processes pose considerable hurdles, especially if relying on fossil fuels for energy, which would negate environmental benefits. Furthermore, the required infrastructure for large-scale DAC deployment, including capture facilities, CO_2_ transport networks, and geological storage sites, demands massive build-out and significant investment, which can be challenging in regions with limited resources. The perceived and real uncertainties surrounding the business cases and economies of scale for commercial operations constrain large private financing flows, making DAC projects risky investments for many. Critics also express concern that DAC development is closely tied to oil and gas interests, potentially distracting from crucial emissions reductions and creating a “predatory delay” in the transition away from fossil fuels.

However, opportunities exist to enhance DAC adoption in developing countries [131]. The technology can be taken over by businesses once profits are apparent, suggesting a need for mechanisms to de-risk early-stage investments. Governments and multilateral agencies must provide financial support to demonstrate reliable performance and gain broader acceptance, potentially through dedicated “DAC funds” that blend public and private capital. The cost of DAC and storage is expected to fall to between USD 100 and 600/t-CO_2_ by 2050 through learning-by-doing rules and mass manufacturing of modular units. Creating market mechanisms and policy interventions to develop demand for Carbon Removal as a Service (CRaaS) and a vibrant carbon market backed by prudent climate action policies can shape DAC technology and drive private capital flows. The adaptability of DAC in resource-limited developing countries is not inherent but rather contingent on significant external support, strategic policy design, and the maturation of the technology to achieve substantial cost reductions and integrate with local renewable energy sources and utilization pathways. This highlights that while DAC is location-flexible in principle, its practical implementation is deeply intertwined with economic and infrastructural development.

## 7. Comparison with Other Carbon Removal and Capture Approaches

DAC is designed to actively take CO_2_ out of the atmosphere and lock it away permanently. This distinguishes it from emissions reduction solutions, which focus on cutting future emissions, and point-source carbon capture technologies, which stop new CO_2_ emissions from being released from an industrial source. Understanding these distinctions is crucial for positioning DAC within the broader climate change mitigation portfolio [152]. As mentioned above, DAC offers several unique advantages. It is location-flexible, meaning it can be built almost anywhere with access to clean energy and CO_2_ storage or reuse options, decoupling carbon removal from emission origins. The technology is scalable, with commercial plants already demonstrating the potential to remove millions of tonnes of CO_2_ from the atmosphere. DAC produces high-purity CO_2_ output, making it ideal for various utilization pathways. It also requires a minimal land footprint compared to nature-based solutions, leading to less disruption to ecosystems. DAC systems are designed to run using renewable electricity, supporting the clean energy transition, and can operate as a continuous process around the clock. However, DAC is currently more expensive than some other carbon removal technologies, and its widespread adoption is reliant on stable, long-term policy support backed by regulation to unlock larger-scale project financing [133].

Point-source carbon capture, often referred to as Carbon Capture and Storage (CCS) or Carbon Capture, Utilization, and Storage (CCUS), is another approach to mitigate climate change, which involves capturing CO_2_ directly from large industrial emitters before it enters the atmosphere. Almost 50% of greenhouse gas emissions in the United States come directly from energy production or industry, making these large “point sources” prime targets for CCS. The primary advantage of CCS is its ability to capture highly concentrated CO_2_ from flue gases, such as those from power plants, cement production, and steel manufacturing. This high concentration makes the capture process less energy-intensive compared to DAC, where CO_2_ is extremely dilute. CCS can abate both process emissions (inherent to chemical reactions) and thermal emissions (from fuel combustion) [153]. Despite these advantages, CCS faces significant disadvantages. Implementing CCS technology in existing industrial and power plants increases the cost of the final product, often requiring subsidies. Critics argue that CCS creates a “moral hazard” by allowing carbon-intensive industries, particularly fossil fuel operations, to continue operating rather than transitioning to cleaner alternatives [154]. Almost all existing CCS projects are tied to “enhanced oil recovery” (EOR), where captured CO_2_ is injected into depleted oil wells to boost oil production, which ultimately leads to more CO_2_ emissions when the extracted oil is burned. The buildout of CCS infrastructure, including massive pipeline networks and underground injection sites, presents serious health, safety, and environmental risks, such as potential CO_2_ leaks, contamination of drinking water, and stimulation of seismic activity. Furthermore, CCS typically only captures a fraction of carbon emissions and does not address other harmful pollutants from fuel combustion. The IPCC’s Sixth Assessment Report indicates that there are no scenarios in which CCUS would allow continued use of fossil fuels at current levels, emphasizing that it is a complementary tool for hard-to-abate sectors, not a license to perpetuate fossil fuel use.

Bioenergy with Carbon Capture and Storage (BECCS) combines the production of energy from biomass with the capture and storage of the CO_2_ emissions produced during the process [155]. In theory, biomass removes CO_2_ from the atmosphere as it grows, and capturing the CO_2_ released during energy conversion could result in “net negative emissions”. This makes BECCS a potentially crucial technology for offsetting emissions from sectors difficult to decarbonize. However, BECCS faces considerable challenges, particularly regarding scalability and environmental impacts. Scaling up BECCS could require vast land areas, potentially leading to land-use conflicts, biodiversity loss, and even increased net emissions in the short-to-medium term if not managed sustainably (e.g., through clear-cutting forests). Studies suggest that after 20 years of operation, the uncaptured emissions from BECCS could be nearly equal to that of a coal plant, as it takes decades for replanted seedlings to absorb enough CO_2_ to make the total lifecycle emissions negative. Supply chain challenges, ensuring sustainable biomass sourcing, and difficulties in measurement and verification also pose hurdles [152].

Afforestation (planting new forests) and reforestation (replanting existing forests) are natural climate solutions that reinforce terrestrial carbon sinks. These methods are relatively cost-effective and contribute to maintaining the water cycle, preventing soil erosion and creating animal habitats [156]. Trees absorb CO_2_ as they grow, storing it in their roots, trunks, and soil, making it one of the best natural ways to reduce greenhouse gases. Despite their benefits, afforestation and reforestation efforts often lack permanence due to risks from droughts, wildfires, tree diseases, and deforestation. While trees absorb carbon immediately, it can take decades for them to absorb significant amounts of CO_2_ (e.g., 10–40 kg per year per tree). This means they do not reduce carbon emissions immediately at the scale and speed required for today’s emissions. Furthermore, planting non-native trees or afforesting in inappropriate biomes (e.g., grasslands) can negatively impact previously established ecosystems and reduce biodiversity. These methods can also be harder to measure, verify, and manage over time compared to technological solutions.

Finally, Industrial decarbonization encompasses a wide array of strategies beyond direct carbon capture [157]. The International Energy Agency (IEA) notes that industrial emissions must decrease by 43% by 2030 (compared to 2019) to meet 2050 net-zero targets. Key strategies include the following: (i) energy efficiency: improving energy use across various industrial processes; (ii) heat electrification: shifting industrial heating processes from fossil fuels to electricity, particularly from renewable sources; (iii) green fuels: transitioning to emission-free fuels like green hydrogen (produced via electrolysis with renewable electricity) as a fossil fuel replacement; (iv) recycling: increasing recycling rates, especially in steel, aluminum, and plastics industries, to reduce demand for raw materials and associated emissions; (v) process optimization and material substitution: redesigning industrial processes to inherently reduce emissions and using lower-carbon materials.

In this broader context, CCS and DAC are viewed as complementary tools rather than standalone solutions. They are particularly relevant for “hard-to-abate” sectors where eliminating fossil fuel use or other emissions is not yet technically or economically feasible. The IPCC’s scenarios emphasize that while carbon capture technologies can contribute to emissions reduction, they do not allow for the continued use of fossil fuels at current levels. The optimal strategy for climate change mitigation involves a diverse portfolio of approaches, combining aggressive emissions reductions across all sectors with various carbon removal methods, each tailored to specific contexts and leveraging their respective advantages while mitigating their limitations.

## 8. Environmentally Friendly Handling of Spent Nanomaterials

The long-term stability of sorbent materials under repeated adsorption–desorption cycles, fluctuating temperatures, and the presence of atmospheric contaminants is crucial for the economic viability and environmental footprint of DAC. Sorbent degradation, such as oxidative degradation, amine leaching, or structural collapse in amine-functionalized polymers and MOFs, leads to a reduction in CO_2_ capture capacity and lifespan. This necessitates frequent sorbent replacement, increasing operational costs and environmental footprint. Inorganic sorbents like zeolites and LDHs generally offer higher thermal stability but may still face issues with hydration/dehydration cycles or poisoning by other atmospheric components over extended operation.

### 8.1. Environmental Concerns of Nanomaterial Production and Disposal

The production and eventual disposal of nanomaterials used in DAC raise several environmental considerations. The synthesis of many advanced nanomaterials, such as MOFs and COFs, often involves the use of strong acids, high temperatures, and toxic solvents. These processes, if not properly managed, can lead to the release of harmful byproducts and volatile organic compounds (VOCs) into the atmosphere, contributing to air pollution and potential harm to ecosystems. Furthermore, the large-scale production of these materials necessitates significant amounts of resources, including metals and organic ligands, whose extraction and processing can have negative environmental impacts such as habitat destruction, water pollution, and high energy consumption [158]. Beyond production, the degradation of sorbents during DAC operation can also pose environmental risks. Degraded sorbents may release unwanted pollutants, and there are concerns about hazardous chemicals contaminating water sources if waste management practices are improper. The entire lifecycle of these materials, from raw material extraction and synthesis to operation and end-of-life disposal, must be carefully considered to ensure the net environmental benefit of DAC [159].

### 8.2. Recycling and Regeneration Strategies

Efficient regeneration is paramount for extending sorbent lifetime and reducing overall costs and environmental impact [46]. Research efforts are focused on developing lower-temperature regeneration methods to reduce degradation, with some coated sorbents demonstrating regeneration at temperatures as low as 50 °C. Direct steam stripping is emerging as a cost-effective and energy-efficient regeneration process for solid sorbents, potentially avoiding sorbent degradation and significantly increasing regeneration rates. For example, solid bis(iminoguanidines) (BIGs) have shown up to 99% CO_2_ recovery through direct steam exposure, with fully regenerated material converting into an aqueous solution that can be easily recycled [159].

Specific recycling and regeneration considerations for different nanomaterial classes include the following:

- MOFs: These materials exhibit high cycling stability and low energy requirements for regeneration. Modular DAC systems are being developed that allow for the flexible replacement of MOF-based sorbents as new materials emerge. Furthermore, research is exploring the upcycling of waste materials, such as polyethylene terephthalate (PET) and heavy-metal-containing industrial wastewater, to produce magnetic MOFs, offering a sustainable solution for waste management and environmental cleanup [160].
- COFs: COF-999, for instance, has demonstrated exceptional stability, retaining full performance over 100 adsorption–desorption cycles in open air. This inherent robustness reduces the frequency of replacement and the associated environmental burden [48].
- Zeolites: Zeolites are known for their regenerability and reusability, which helps reduce their overall environmental impact. Efforts are also underway to convert industrial by-products, such as coal fly ash, into synthetic zeolites for use in flue gas cleaning systems, addressing both waste management and carbon capture. Life cycle assessment (LCA) studies indicate that zeolites can have lower environmental loads compared to carbon molecular sieves in carbon capture processes, particularly when renewable energy is used for regeneration [161].
- Polymer-Based Sorbents: These materials are designed for satisfactory performance stability toward regeneration. For economic viability, polymer sorbents are targeted to withstand approximately 100,000 adsorption–desorption cycles, necessitating robust design to minimize degradation over time [162].
- Porous Carbons: Research focuses on upcycling solid waste into porous carbons for CO_2_ capture, which offers dual environmental benefits by mitigating climate change and addressing solid waste management challenges. Captured CO_2_ can also be mineralized with enriched porous carbons for use as an additive in concrete production, providing a viable path for permanent CO_2_ storage in value-added products [163].
- Ionic Liquids: ILs are noted for their high chemical and thermal stability, non-flammability, and tunable properties, making them promising “greener” alternatives to conventional solvents. Efforts are ongoing to reduce the costs and environmental emissions associated with IL-based capture processes through systematic selection and heat integration [109].

### 8.3. Life Cycle Assessment of DAC

A comprehensive life cycle assessment (LCA) is crucial for evaluating the true environmental performance of DACCS systems. Studies reveal substantial variability in life cycle efficiency and environmental impacts across different DACCS configurations [135]. Solid sorbent technologies demonstrate average net greenhouse gas reductions of 640 kg CO_2_-eq/t CO_2_, while liquid sorbent systems achieve reductions of about 560 kg CO_2_-eq/t CO_2_, with system carbon efficiencies ranging between 56% and 64% [135]. Beyond climate impacts, DACCS systems exhibit significant resource demands, with water consumption ranging from 1 to 12 tons per ton of CO_2_ captured, and land use spanning 85–4450 km^2^ depending on system configuration and renewable energy requirements [152]. For gigaton-scale facilities, significant environmental trade-offs emerge, including substantial particulate matter emissions (170–180 kt annually) and varying impacts on marine eutrophication (up to 90% higher for amine-based systems compared to hydroxide-based alternatives). Low-temperature DAC systems may exhibit higher human toxicity and ecotoxicity impacts due to increased electricity demands, while metal resource depletion varies significantly based on system design and energy sources. A critical finding from LCA is the importance of selecting appropriate locations for grid-coupled system layouts, as deploying DACCS at geographic locations with CO_2_-intensive grid electricity mixes can lead to net GHG emissions instead of GHG removal [152]. This underscores that the environmental benefits of DAC are highly dependent on the energy source used for its operation, particularly for regeneration, and the overall resource footprint of the materials throughout their lifecycle. Developing nanomaterials with robust hydrophobic surfaces or selective water exclusion mechanisms and ensuring the robustness of nanomaterial structures and functional groups under real-world DAC conditions are key research priorities to extend sorbent lifetime and reduce the overall environmental burden.

## 9. Conclusions

Direct air capture (DAC) is a critical technology in the fight against climate change, offering a way to remove CO_2_ directly from the atmosphere and contribute to global decarbonization efforts. This review has provided a comprehensive overview of the state-of-the-art materials used for DAC, exploring various classes of sorbents, including solid sorbents like metal–organic frameworks (MOFs), covalent organic frameworks (COFs), zeolites, amine-functionalized polymers, porous carbons, and layered double hydroxides (LDHs), as well as solid-supported ionic liquids and emerging technologies such as Moisture Swing Adsorption (MSA), Electro Swing Adsorption (ESA), Passive DAC, and CO_2_-Binding Organic Liquids (CO_2_ BOLs).

Solid sorbents, with their high surface area, tunable pore structures, and potential for selective CO_2_ adsorption, have emerged as promising candidates for DAC applications, with their nanoscale properties being fundamental to their performance. MOFs, in particular, have garnered significant attention due to their exceptional CO_2_ capture capacity, selectivity, and relatively low regeneration temperatures, although their high cost and water sensitivity remain challenges. Zeolites offer high thermal and chemical stability and cost-effectiveness, but their inherent hydrophilicity can reduce efficiency in humid conditions. Amine-functionalized polymers present high CO_2_ uptake capacity and maintain performance under humid conditions, but their thermal stability and regeneration energy require further optimization. Carbonaceous materials offer low cost and high stability but struggle with low CO_2_ selectivity in dilute air. LDHs provide low cost and energy-efficient regeneration but have limited capacity and long-term stability concerns under fluctuating conditions. Solid-supported ionic liquids offer tunable chemistry and negligible vapor pressure, but high viscosity and production costs are notable limitations [164].

Moisture swing adsorption (MSA) stands out as a promising DAC technology that leverages ambient humidity to drive CO_2_ capture and release, offering potential for reduced energy consumption and cost-effectiveness compared to conventional temperature or pressure swing adsorption processes. However, its absolute reliance on specific humidity conditions introduces operational complexities that must be meticulously managed. Emerging DAC technologies like ESA, Passive DAC, and CO_2_ BOLs hold significant potential for further improving efficiency and cost-effectiveness, with many integrating nanostructured components to enhance performance [165].

A critical aspect of this comprehensive review has been addressing the real market for DAC and its practical applications. The global DAC market is projected for substantial growth, driven by net-zero targets, corporate commitments, and technological advancements. DAC finds diverse applications across energy, transportation, industrial, and agricultural sectors, with leading companies actively developing and deploying solutions. However, the adaptability of DAC for developing countries with limited resources faces significant hurdles, primarily due to high costs, energy demands, and infrastructure limitations. Successful deployment in these regions will necessitate substantial external investment, strategic policy frameworks, and the maturation of technologies to achieve significant cost reductions.

The comparison with other carbon removal and capture approaches highlights that DAC is not a singular solution but a crucial component within a broader climate mitigation portfolio. Unlike point-source carbon capture, which addresses concentrated emissions, DAC targets diffuse atmospheric CO_2_ and is location-flexible. While natural solutions like afforestation are cost-effective, they often lack permanence and are slow to act. Bioenergy with Carbon Capture and Storage (BECCS) offers theoretical net-negative emissions but faces significant scalability and land-use challenges. The optimal path to climate stabilization requires a diverse portfolio, combining aggressive emissions reductions across all sectors with various carbon removal methods, each strategically deployed based on regional factors and resource availability.

Despite the significant progress in DAC technologies, several formidable challenges remain, including high capital costs, substantial energy demands, water sensitivity, and scalability issues. Sorbent degradation over repeated cycles also poses a critical operational and environmental concern. The environmentally friendly handling of spent nanomaterials necessitates a holistic approach, considering the environmental impacts from synthesis (e.g., use of strong acids, toxic solvents) to operational degradation and end-of-life management. Research is actively pursuing advanced regeneration strategies, such as lower-temperature methods and direct steam stripping, and exploring the recycling and upcycling of spent sorbents, including MOFs, zeolites, and porous carbons, into value-added products. Life Cycle Assessments (LCAs) underscore the variability in environmental impact across different DAC systems, emphasizing the critical role of energy source and resource consumption in determining net climate benefits. Furthermore, while CO_2_ utilization pathways can enhance economic viability, a significant maturity mismatch currently exists between DAC and most utilization techniques, requiring simultaneous investment and supportive policy frameworks to bridge this gap [166].

Overcoming these challenges requires continued research and development across multiple domains, including the design of new, more robust, and water-resistant nanomaterial sorbents, optimization of DAC processes, seamless integration with complementary technologies, and supportive policy frameworks to incentivize innovation and accelerate deployment. The development and deployment of DAC technologies are crucial for achieving global climate goals. As the technology matures, DAC is poised to play an increasingly vital role in mitigating climate change and enabling the transition to a net-zero carbon future. By effectively addressing the extant challenges and capitalizing on emerging opportunities, DAC can become a truly viable and scalable solution for atmospheric CO_2_ removal, contributing significantly to a sustainable future for generations to come.

## Data Availability

Data will be made available on request.

## References

[1] IPCC 2018 Global Warming of 1.5 °C (2018). An IPCC Special Report on the Impacts of Global Warming of 1.5 °C Above Pre-Industrial Levels and Related Global Greenhouse Gas Emission Pathways, in the Context of Strengthening the Global Response to the Threat of Climate Change.

[2] Hoegh-Guldberg O., Jacob D., Taylor M. (2018). Impacts of 1.5 C Global Warming on Natural and Human Systems; Global Warming of 1.5 C: An IPCC Special Report. Am. J. Epidemiol..

[3] Doney S.C., Fabry V.J., Feely R.A., Kleypas J.A. (2009). Ocean Acidification: The Other CO_2_ Problem. Ann. Rev. Mar. Sci..

[4] Fawzy S., Osman A.I., Doran J., Rooney D.W. (2020). Strategies for Mitigation of Climate Change: A Review. Environ. Chem. Lett..

[5] Griscom B.W., Adams J., Ellis P.W., Houghton R.A., Lomax G., Miteva D.A., Schlesinger W.H., Shoch D., Siikamäki J.V., Smith P. (2017). Natural Climate Solutions. Proc. Natl. Acad. Sci. USA.

[6] National Academies of Sciences, Engineering, and Medicine (2019). Negative Emissions Technologies and Reliable Sequestration: A Research Agenda.

[7] Masson-Delmotte V., Zhai P., Pirani A., Connors S.L., Péan C., Berger S., Caud N., Chen Y., Goldfarb L., Gomis M.I. Climate Change 2021: The Physical Science Basis. Contribution of Working Group I to the Sixth Assessment Report of the Intergovernmental Panel on Climate Change. Proceedings of the IPCC 2021.

[8] Fuss S., Canadell J.G., Peters G.P., Tavoni M., Andrew R.M., Ciais P., Jackson R.B., Jones C.D., Kraxner F., Nakicenovic N. (2014). Betting on Negative Emissions. Nat. Clim. Change.

[9] Sanz-Pérez E.S., Murdock C.R., Didas S.A., Jones C.W. (2016). Direct Capture of CO_2_ from Ambient Air. Chem. Rev..

[10] Keith D.W., Holmes G., St. Angelo D., Heidel K. (2018). A Process for Capturing CO_2_ from the Atmosphere. Joule.

[11] Socolow R., Desmond M., Aines R., Blackstock J., Bolland O., Kaarsberg T., Lewis N., Mazzotti M., Pfeffer A., Sawyer K. Direct Air Capture of CO_2_ with Chemicals A Technology Assessment for the APS Panel on Public Affairs. *Technology*
**2011**, 1–119. https://carbonengineering.com/wp-content/uploads/2019/11/APS_DAC_Report-FINAL_Original.pdf.

[12] Lackner K.S. (2003). A Guide to CO_2_ Sequestration. Science.

[13] Sedjo R., Sohngen B. (2012). Carbon Sequestration in Forests and Soils. Annu. Rev. Resour. Econ..

[14] Davis S.J., Caldeira K., Matthews H.D. (2011). Future CO_2_ Emissions and Climate Change from Existing Energy Infrastructure. Science.

[15] Hansen J., Sato M., Kharecha P., Beerling D., Berner R., Masson-Delmotte V., Pagani M., Raymo M., Royer D.L., Zachos J.C. (2008). Target Atmospheric CO_2_: Where Should Humanity Aim?. Open Atmos. Sci. J..

[16] Boot-Handford M.E., Abanades J.C., Anthony E.J., Blunt M.J., Brandani S., Mac Dowell N., Fernández J.R., Ferrari M.C., Gross R., Hallett J.P. (2014). Carbon Capture and Storage Update. Energy Environ. Sci..

[17] Sun S., Sun H., Williams P.T., Wu C. (2021). Recent Advances in Integrated CO_2_ Capture and Utilization: A Review. Sustain. Energy Fuels.

[18] Fasihi M., Efimova O., Breyer C. (2019). Techno-Economic Assessment of CO_2_ Direct Air Capture Plants. J. Clean. Prod..

[19] Holmes G., Keith D.W. (2012). An Air-Liquid Contactor for Large-Scale Capture of CO_2_ from Air. Philos. Trans. R. Soc. A Math. Phys. Eng. Sci..

[20] Choi S., Drese J.H., Jones C.W. (2009). Adsorbent Materials for Carbon Dioxide Capture from Large Anthropogenic Point Sources. ChemSusChem.

[21] Rochelle G.T. (2009). Amine Scrubbing for CO_2_ Capture. Geol. Nat. Phys. Rev. Proc. R. Soc. London Ser. A Math. Phys. Sci. Carbon Capture J..

[22] Didas S.A., Kulkarni A.R., Sholl D.S., Jones C.W. (2012). Role of Amine Structure on Carbon Dioxide Adsorption from Ultradilute Gas Streams Such as Ambient Air. ChemSusChem.

[23] Heldebrant D.J., Koech P.K., Glezakou V.A., Rousseau R., Malhotra D., Cantu D.C. (2017). Water-Lean Solvents for Post-Combustion CO_2_ Capture: Fundamentals, Uncertainties, Opportunities, and Outlook. Chem. Rev..

[24] Lepaumier H., Picq D., Carrette P.L. (2009). New Amines for CO_2_ Capture. II. Oxidative Degradation Mechanisms. Ind. Eng. Chem. Res..

[25] Veltman K., Singh B., Hertwich E.G. (2010). Human and Environmental Impact Assessment of Postcombustion CO_2_ Capture Focusing on Emissions from Amine-Based Scrubbing Solvents to Air. Environ. Sci. Technol..

[26] Sen R., Goeppert A., Kar S., Prakash G.K.S. (2020). Hydroxide Based Integrated CO_2_ Capture from Air and Conversion to Methanol. J. Am. Chem. Soc..

[27] Sen R., Koch C.J., Galvan V., Entesari N., Goeppert A., Prakash G.K.S. (2021). Glycol Assisted Efficient Conversion of CO_2_ Captured from Air to Methanol with a Heterogeneous Cu/ZnO/Al_2_O_3_ Catalyst. J. CO_2_ Util..

[28] Brethomé F.M., Williams N.J., Seipp C.A., Kidder M.K., Custelcean R. (2018). Direct Air Capture of CO_2_ via Aqueous-Phase Absorption and Crystalline-Phase Release Using Concentrated Solar Power. Nat. Energy.

[29] IEA (2022). Direct Air Capture: A Key Technology for Net Zero.

[30] Hanusch J.M., Kerschgens I.P., Huber F., Neuburger M., Gademann K. (2019). Pyrrolizidines for Direct Air Capture and CO_2_ Conversion. Chem. Commun..

[31] Inagaki F., Matsumoto C., Iwata T., Mukai C. (2017). CO_2_-Selective Absorbents in Air: Reverse Lipid Bilayer Structure Forming Neutral Carbamic Acid in Water without Hydration. J. Am. Chem. Soc..

[32] Barzagli F., Giorgi C., Mani F., Peruzzini M. (2020). Screening Study of Different Amine-Based Solutions as Sorbents for Direct CO_2_ Capture from Air. ACS Sustain. Chem. Eng..

[33] Custelcean R., Williams N.J., Garrabrant K.A., Agullo P., Brethomé F.M., Martin H.J., Kidder M.K. (2019). Direct Air Capture of CO_2_ with Aqueous Amino Acids and Solid Bis-Iminoguanidines (BIGs). Ind. Eng. Chem. Res..

[34] Seipp C.A., Williams N.J., Kidder M.K., Custelcean R. (2017). CO_2_ Capture from Ambient Air by Crystallization with a Guanidine Sorbent. Angew. Chemie Int. Ed..

[35] Sumida K., Rogow D.L., Mason J.A., McDonald T.M., Bloch E.D., Herm Z.R., Bae T.H., Long J.R. (2012). Carbon Dioxide Capture in Metal-Organic Frameworks. Chem. Rev..

[36] Li J.R., Kuppler R.J., Zhou H.C. (2009). Selective Gas Adsorption and Separation in Metal-Organic Frameworks. Chem. Soc. Rev..

[37] Burtch N.C., Jasuja H., Walton K.S. (2014). Water Stability and Adsorption in Metal-Organic Frameworks. Chem. Rev..

[38] Wang W., Liu F., Zhang Q., Yu G., Deng S. (2020). Efficient Removal of CO_2_ from Indoor Air Using a Polyethyleneimine-Impregnated Resin and Its Low-Temperature Regeneration. Chem. Eng. J..

[39] Lee W.R., Hwang S.Y., Ryu D.W., Lim K.S., Han S.S., Moon D., Choi J., Hong C.S. (2014). Diamine-Functionalized Metal-Organic Framework: Exceptionally High CO_2_ Capacities from Ambient Air and Flue Gas, Ultrafast CO_2_ Uptake Rate, and Adsorption Mechanism. Energy Environ. Sci..

[40] Zanatta M. (2023). Materials for Direct Air Capture and Integrated CO_2_ Conversion: Advancement, Challenges, and Prospects. ACS Mater. Au.

[41] Sodiq A., Abdullatif Y., Aissa B., Ostovar A., Nassar N., El-Naas M., Amhamed A. (2023). A Review on Progress Made in Direct Air Capture of CO_2_. Environ. Technol. Innov..

[42] Boone P., He Y., Lieber A.R., Steckel J.A., Rosi N.L., Hornbostel K.M., Wilmer C.E. (2022). Designing Optimal Core-Shell MOFs for Direct Air Capture. Nanoscale.

[43] Lin K.Y., Xie Z.M., Hong L.S., Jiang J.C. (2023). Insights into the Capture Mechanism of CO_2_ by Diamine-Appended Mg2(Dobpdc): A Combined DFT and Microkinetic Modeling Study. J. Mater. Chem. C.

[44] Chen O.I.F., Liu C.H., Wang K., Borrego-Marin E., Li H., Alawadhi A.H., Navarro J.A.R., Yaghi O.M. (2024). Water-Enhanced Direct Air Capture of Carbon Dioxide in Metal-Organic Frameworks. J. Am. Chem. Soc..

[45] Sadiq M.M., Batten M.P., Mulet X., Freeman C., Konstas K., Mardel J.I., Tanner J., Ng D., Wang X., Howard S. (2020). A Pilot-Scale Demonstration of Mobile Direct Air Capture Using Metal-Organic.Pdf. Adv. Sustain. Syst..

[46] An K., Li K., Yang C.M., Brechtl J., Nawaz K. (2023). A Comprehensive Review on Regeneration Strategies for Direct Air Capture. J. CO_2_ Util..

[47] Azarabadi H., Lackner K.S. (2019). A Sorbent-Focused Techno-Economic Analysis of Direct Air Capture. Appl. Energy.

[48] Zhou Z., Ma T., Zhang H., Chheda S., Li H., Wang K., Ehrling S., Giovine R., Li C., Alawadhi A.H. (2024). Carbon Dioxide Capture from Open Air Using Covalent Organic Frameworks. Nature.

[49] Institute W.R. 6 Things to Know About Direct Air Capture. https://www.wri.org/insights/direct-air-capture-resource-considerations-and-costs-carbon-removal.

[50] Xiang X., Guo T., Yin Y., Gao Z., Wang Y., Wang R., An M., Guo Q., Hu X. (2023). High Adsorption Capacity Fe@13X Zeolite for Direct Air CO_2_ Capture. Ind. Eng. Chem. Res..

[51] Fu D., Davis M.E. (2023). Toward the Feasible Direct Air Capture of Carbon Dioxide with Molecular Sieves by Water Management. Cell Rep. Phys. Sci..

[52] Stuckert N.R., Yang R.T. (2011). CO_2_ Capture from the Atmosphere and Simultaneous Concentration Using Zeolites and Amine-Grafted SBA-15. Environ. Sci. Technol..

[53] Wang Y., Jia H., Chen P., Fang X., Du T. (2020). Synthesis of La and Ce Modified X Zeolite from Rice Husk Ash for Carbon Dioxide Capture. J. Mater. Res. Technol..

[54] Wilson S.M.W. (2022). The Potential of Direct Air Capture Using Adsorbents in Cold Climates. Iscience.

[55] Kumar R., Ohtani S., Tsunoji N. (2023). Direct Air Capture on Amine-Impregnated FAU Zeolites: Exploring for High Adsorption Capacity and Low-Temperature Regeneration. Microporous Mesoporous Mater..

[56] Wilson S.M.W., Tezel F.H. (2020). Direct Dry Air Capture of CO_2_ Using VTSA with Faujasite Zeolites. Ind. Eng. Chem. Res..

[57] Su F., Lu C., Kuo S.C., Zeng W. (2010). Adsorption of CO_2_ on amine-functionalized y-type zeolites. Energy and Fuels.

[58] Davarpanah E., Armandi M., Hernández S., Fino D., Arletti R., Bensaid S., Piumetti M. (2020). CO_2_ Capture on Natural Zeolite Clinoptilolite: Effect of Temperature and Role of the Adsorption Sites. J. Environ. Manag..

[59] S Alivand M., Habiba U., Ghasemian M., Askari S., Webley P.A. (2024). Amine-Functionalized Meso-Macroporous Polymers for Efficient CO_2_ Capture from Ambient Air. ACS Appl. Mater. Interfaces.

[60] Samaddoost L., Soltani M., Fatehifar E., Abbasi Asl E. (2023). Design of Amine-Functionalized Resin via a Facial Method with Efficient CO_2_ Capture from Air. Process Saf. Environ. Prot..

[61] Sekizkardes A.K., Kusuma V.A., Culp J.T., Muldoon P., Hoffman J., Steckel J.A., Hopkinson D. (2023). Single Polymer Sorbent Fibers for High Performance and Rapid Direct Air Capture. J. Mater. Chem. A.

[62] Chen Z., Deng S., Wei H., Wang B., Huang J., Yu G. (2013). Polyethylenimine-Impregnated Resin for High CO_2_ Adsorption: An Efficient Adsorbent for CO_2_ Capture from Simulated Flue Gas and Ambient Air. ACS Appl. Mater. Interfaces.

[63] Yang M., Wang S., Xu L. (2023). Hydrophobic Functionalized Amine-Impregnated Resin for CO_2_ Capture in Humid Air. Sep. Purif. Technol..

[64] Zeeshan M., Kidder M.K., Pentzer E., Getman R.B., Gurkan B. (2023). Direct Air Capture of CO_2_: From Insights into the Current and Emerging Approaches to Future Opportunities. Front. Sustain..

[65] Carbon Engineering (2025). Research Round-Up: Evaluating Direct Air Capture Pathways.

[66] Priyadarshini P., Rim G., Rosu C., Song M.G., Jones C.W. (2023). Direct Air Capture of CO_2_ Using Amine/Alumina Sorbents at Cold Temperature. ACS Environ. Au.

[67] Zhang B., Jiang Y., Balasubramanian R. (2022). Synthesis of Biowaste-Derived Carbon Foam for CO_2_ Capture. Resour. Conserv. Recycl..

[68] Climeworks (2025). Carbon removal solutions for your net zero targets. https://climeworks.com/.

[69] National Energy Technology Laboratory (2025). A one-of-a-kind facility supporting rapid technology development for atmospheric carbon capture. https://netl.doe.gov/dac.

[70] Wang Q., Yan Q., Zhao Y., Ren J., Ai N. (2022). Preparation of Amine-Modified Cu-Mg-Al Ldh Composite Photocatalyst. Nanomaterials.

[71] Ge B., Chen C., Gan Z., Zhu X., Miao Y., Wang Y., Ge T., O’Hare D., Wang R. (2023). Scalable Synthesis of Amine-Grafted Ultrafine Layered Double Hydroxide Nanosheets with Improved Carbon Dioxide Capture Capacity from Air. ACS Sustain. Chem. Eng..

[72] Zhu X., Ge T., Yang F., Lyu M., Chen C., O’Hare D., Wang R. (2020). Efficient CO_2_ Capture from Ambient Air with Amine-Functionalized Mg-Al Mixed Metal Oxides. J. Mater. Chem. A.

[73] Zhao M., Xiao J., Gao W., Wang Q. (2022). Defect-Rich Mg-Al MMOs Supported TEPA with Enhanced Charge Transfer for Highly Efficient and Stable Direct Air Capture. J. Energy Chem..

[74] Zhang Y., Ding L., Xie Z., Zhang X., Sui X., Li J.-R. (2025). Porous Sorbents for Direct Capture of Carbon Dioxide from Ambient Air. Chin. Chem. Lett..

[75] Phil De Luna Will Direct Air Capture Ever Cost Less than $100 per Ton of CO_2_? *Illuminen* 2024. https://illuminem.com/illuminemvoices/will-direct-air-capture-ever-cost-less-than-100-per-ton-of-co.

[76] Rim G., Song M., Proaño L., Ghaffari Nik O., Parker S., Lively R.P., Jones C.W. (2025). Humidity Effects on Sub-Ambient Direct Air Capture of CO_2_ with Amine Functionalized Mg-Al LDHs and MMOs. ACS ES T Eng..

[77] Chen Y., Ji G., Guo S., Yu B., Zhao Y., Wu Y., Zhang H., Liu Z., Han B., Liu Z. (2017). Visible-Light-Driven Conversion of CO_2_ from Air to CO Using an Ionic Liquid and a Conjugated Polymer. Green Chem..

[78] Zeeshan M., Klemm A., Damron J.T., Unocic K.A., Kidder M.K., Gurkan B. (2024). Ionic Liquid Functionalizes the Metal Organic Framework for Microwave-Assisted Direct Air Capture of CO_2_. ACS Mater. Lett..

[79] Taylor C.D.L., Klemm A., Al-Mahbobi L., Bradford B.J., Gurkan B., Pentzer E.B. (2024). Ionic Liquid-Glycol Mixtures for Direct Air Capture of CO_2_: Decreased Viscosity and Mitigation of Evaporation via Encapsulation. ACS Sustain. Chem. Eng..

[80] Heldebrant D.J., Yonker C.R., Jessop P.G., Phan L. (2009). CO_2_—Binding Organic Liquids (CO_2_ BOLs) for Post-Combustion CO_2_ Capture. Energy Procedia.

[81] Zhang X., Zhao H., Yang Q., Yao M., Wu Y.N., Gu Y. (2023). Direct Air Capture of CO_2_ in Designed Metal-Organic Frameworks at Lab and Pilot Scale. Carbon Capture Sci. Technol..

[82] Fracaroli A.M., Furukawa H., Suzuki M., Dodd M., Okajima S., Gándara F., Reimer J.A., Yagh O.M. (2014). Metal-Organic Frameworks with Precisely Designed Interior for Carbon Dioxide Capture in the Presence of Water. J. Am. Chem. Soc..

[83] Gopalsamy K., Fan D., Naskar S., Magnin Y., Maurin G. (2024). Engineering of an Isoreticular Series of CALF-20 Metal-Organic Frameworks for CO_2_ Capture. ACS Appl. Eng. Mater..

[84] Rohde R.C., Carsch K.M., Dods M.N., Jiang H.Z.H., McIsaac A.R., Klein R.A., Kwon H., Karstens S.L., Wang Y., Huang A.J. (2024). High-Temperature Carbon Dioxide Capture in a Porous Material with Terminal Zinc Hydride Sites. Science.

[85] Chung Y.G., Gómez-Gualdrón D.A., Li P., Leperi K.T., Deria P., Zhang H., Vermeulen N.A., Stoddart J.F., You F., Hupp J.T. (2016). In Silico Discovery of Metal-Organic Frameworks for Precombustion CO_2_ Capture Using a Genetic Algorithm. Sci. Adv..

[86] Cammarere C., Cortés J., Glover T.G., Snurr R.Q., Hupp J.T., Liu J. (2025). Water-Enhanced CO_2_ Capture in Metal-Organic Frameworks. Front. Chem..

[87] Su F., Lu C. (2012). CO_2_ Capture from Gas Stream by Zeolite 13X Using a Dual-Column Temperature/Vacuum Swing Adsorption. Energy Environ. Sci..

[88] Li G., Xiao P., Webley P., Zhang J., Singh R., Marshall M. (2008). Capture of CO_2_ from High Humidity Flue Gas by Vacuum Swing Adsorption with Zeolite 13X. Adsorption.

[89] Mohamed M.G., El-Mahdy A.F.M., Kotp M.G., Kuo S.W. (2022). Advances in Porous Organic Polymers: Syntheses, Structures, and Diverse Applications. Mater. Adv..

[90] Nicotera I., Policicchio A., Conte G., Agostino R.G., Lufrano E., Simari C. (2022). Quaternary Ammonium-Functionalized Polysulfone Sorbent: Toward a Selective and Reversible Trap-Release of CO_2_. J. CO_2_ Util..

[91] Nicotera I., Policicchio A., Conte G., Giuseppe R., Habib M., Rehman U., Lufrano E., Simari C. (2022). Quaternized Polyepichlorohydrin-Based Membrane as High-Selective CO_2_ Sorbent for Cost-Effective Carbon Capture. J. CO_2_ Util..

[92] Liang T., Chen C., Li X., Zhang J. (2016). Popcorn-Derived Porous Carbon for Energy Storage and CO_2_ Capture. Langmuir.

[93] Li Y., Wang X., Cao M. (2018). Three-Dimensional Porous Carbon Frameworks Derived from Mangosteen Peel Waste as Promising Materials for CO_2_ Capture and Supercapacitors. J. CO_2_ Util..

[94] Bari G.A.K.M.R., Jeong J.H. (2023). Porous Carbon for CO_2_ Capture Technology: Unveiling Fundamentals and Innovations. Surfaces.

[95] Tian K., Wu Z., Xie F., Hu W., Li L. (2017). Nitrogen-Doped Porous Carbons Derived from Triarylisocyanurate-Cored Polymers with High CO_2_ Adsorption Properties. Energy Fuels.

[96] Joseph S., Singh G., Lee J.M., Yu X., Breese M.B., Ruban S.M., Bhargava S.K., Yi J., Vinu A. (2023). Hierarchical Carbon Structures from Soft Drink for Multi-Functional Energy Applications of Li-Ion Battery, Na-Ion Battery and CO_2_ Capture. Carbon.

[97] Zhang C., Sun S., He S., Wu C. (2022). Direct Air Capture of CO_2_ by KOH-Activated Bamboo Biochar. J. Energy Inst..

[98] Li R., Hu X., Huang L., Musyoka N.M., Xue T., Wang Q. (2024). Dynamic Intermediate-Temperature CO_2_ Adsorption Performance of K2CO3-Promoted Layered Double Hydroxide-Derived Adsorbents. Molecules.

[99] Chaillot D., Folliard V., Mieh-Brendlé J., Auroux A., Dzene L., Bennici S. (2023). Basic Properties of MgAl-Mixed Oxides in CO_2_ Adsorption at High Temperature. Mater..

[100] Kameda T., Nagano S., Kumagai S., Saito Y., Yoshioka T. (2023). Enrichment of Carbon Dioxide Using Mg-Al Layered Double Hydroxides. Chem. Eng. Res. Des..

[101] Shukla S.K., Khokarale S.G., Bui T.Q., Mikkola J.P.T. (2019). Ionic Liquids: Potential Materials for Carbon Dioxide Capture and Utilization. Front. Mater..

[102] Hospital-Benito D., Moya C., Gazzani M., Palomar J. (2023). Direct Air Capture Based on Ionic Liquids: From Molecular Design to Process Assessment. Chem. Eng. J..

[103] Recker E.A., Green M., Soltani M., Paull D.H., Mcmanus G.J., Davis J.H., Mirjafari A. (2022). Direct Air Capture of CO_2_ via Ionic Liquids Derived from “Waste” Amino Acids. ACS Sustain. Chem. Eng..

[104] Zanatta M., García-Verdugo E., Sans V. (2023). Direct Air Capture and Integrated Conversion of Carbon Dioxide into Cyclic Carbonates with Basic Organic Salts. ACS Sustain. Chem. Eng..

[105] Foorginezhad S., Yu G., Ji X. (2022). Reviewing and Screening Ionic Liquids and Deep Eutectic Solvents for Effective CO_2_ Capture. Front. Chem..

[106] Zhang R., Ke Q., Zhang Z., Zhou B., Cui G., Lu H. (2022). Tuning Functionalized Ionic Liquids for CO_2_ Capture. Int. J. Mol. Sci..

[107] Ab Rahim A.H., Yunus N.M., Bustam M.A. (2023). Ionic Liquids Hybridization for Carbon Dioxide Capture: A Review. Molecules.

[108] Numpilai T., Pham L.K.H., Witoon T. (2024). Advances in Ionic Liquid Technologies for CO_2_ Capture and Conversion: A Comprehensive Review. Ind. Eng. Chem. Res..

[109] Faisal Elmobarak W., Almomani F., Tawalbeh M., Al-Othman A., Martis R., Rasool K. (2023). Current Status of CO_2_ Capture with Ionic Liquids: Development and Progress. Fuel.

[110] Wang T., Lackner K.S., Wright A.B. (2013). Moisture-Swing Sorption for Carbon Dioxide Capture from Ambient Air: A Thermodynamic Analysis. Phys. Chem. Chem. Phys..

[111] Wang T., Lackner K.S., Wright A. (2011). Moisture Swing Sorbent for Carbon Dioxide Capture from Ambient Air. Environ. Sci. Technol..

[112] Zhu Y., Booth A., Hatzell K.B. (2024). Confinement Effects on Moisture-Swing Direct Air Capture. Environ. Sci. Technol. Lett..

[113] Alexandratos S.D. (2009). Ion-Exchange Resins: A Retrospective from Industrial and Engineering Chemistry Research. Ind. Eng. Chem. Res..

[114] He H., Li W., Zhong M., Konkolewicz D., Wu D., Yaccato K., Rappold T., Sugar G., David N.E., Matyjaszewski K. (2013). Reversible CO_2_ Capture with Porous Polymers Using the Humidity Swing. Energy Environ. Sci..

[115] He H., Li W., Lamson M., Zhong M., Konkolewicz D., Hui C.M., Yaccato K., Rappold T., Sugar G., David N.E. (2014). Porous Polymers Prepared via High Internal Phase Emulsion Polymerization for Reversible CO_2_ Capture. Polym..

[116] Jang G.G., Kasturi A., Stamberga D., Custelcean R., Keum J.K., Yiacoumi S., Tsouris C. (2023). Ultra-Fast Microwave Regeneration of CO_2_ Solid Sorbents for Energy-Efficient Direct Air Capture. Sep. Purif. Technol..

[117] Hegarty J., Shindel B., Sukhareva D., Barsoum M.L., Farha O.K., Dravid V. (2023). Expanding the Library of Ions for Moisture-Swing Carbon Capture. Environ. Sci. Technol..

[118] Song J., Zhu L., Shi X., Liu Y., Xiao H., Chen X. (2019). Moisture Swing Ion-Exchange Resin-PO4 Sorbent for Reversible CO_2_ Capture from Ambient Air. Energy Fuels.

[119] He H., Zhong M., Konkolewicz D., Yacatto K., Rappold T., Sugar G., David N.E., Gelb J., Kotwal N., Merkle A. (2013). Three-Dimensionally Ordered Macroporous Polymeric Materials by Colloidal Crystal. Adv. Funct. Mater..

[120] Biery A.R., Shokrollahzadeh Behbahani H., Green M.D., Knauss D.M. (2024). Polydiallylammonium-Polysulfone Multiblock Copolymers for Moisture-Swing Direct Air Capture of Carbon Dioxide. ACS Appl. Polym. Mater..

[121] Nicotera I., Enotiadis A., Simari C. (2024). Quaternized Graphene for High-Performance Moisture Swing Direct Air Capture of CO_2_. Small.

[122] Wang Y., Kim J., Marreiros J., Rangnekar N., Yuan Y., Johnson J.R., McCool B.A., Realff M.J., Lively R.P. (2025). Investigation of Moisture Swing Adsorbents for Direct Air Capture by Dynamic Breakthrough Studies. ACS Sustain. Chem. Eng..

[123] Mapstone G., Kamsma T.M., Xu Z., Jones P.K., Lee A.A., Temprano I., Lee J., De Volder M.F.L., Forse A.C. (2025). Understanding the Mechanism of Electrochemical CO_2_ Capture by Supercapacitive Swing Adsorption. ACS Nano.

[124] Taylor C. Verdox $80m Fundraising Shows Thirst For Direct Carbon Removal Technology. https://www.verdantix.com/insights/blog/verdox-80m-fundraising-shows-thirst-for-direct-carbon-removal-technology#:~:text=Verdox%2C%20an%20electric%20carbon%20capture,Prelude%20Ventures%2C%20and%20Lowercarbon%20Capital.

[125] Wallace B., Rothenberger P. Deep Sky and Carbon Atlantis to Deploy Direct Air Capture (DAC) in Canada. https://www.deepskyclimate.com/blog/deep-sky-and-carbon-atlantis-to-deploy-direct-air-capture-dac-in-canada.

[126] Lackner K.S., Brennan S., Matter J.M., Park A.H.A., Wright A., Van Der Zwaan B. (2012). The Urgency of the Development of CO_2_ Capture from Ambient Air. Proc. Natl. Acad. Sci. USA.

[127] Ho M., Barahimi V., Croiset E. (2025). The State of Direct Air Capture Technology and Industry DIRECT AIR CAPTURE.

[128] Heldebrant D.J., Koech P.K., Ang M.T.C., Liang C., Rainbolt J.E., Yonker C.R., Jessop P.G. (2010). Reversible Zwitterionic Liquids, the Reaction of Alkanol Guanidines, Alkanol Amidines, and Diamines with CO_2_. Green Chem..

[129] Breyer C., Fasihi M., Bajamundi C., Creutzig F. (2019). Direct Air Capture of CO_2_: A Key Technology for Ambitious Climate Change Mitigation. Joule.

[130] Li J., Bilal M., Landskron K. (2024). Scaling Supercapacitive Swing Adsorption of CO_2_ Using Bipolar Electrode Stacks. Small.

[131] Koch R., Dittmeyer R. (2025). Comparative Analysis of Industrialization Potentials of Direct Air Capture Technologies. Front. Clim..

[132] Bouaboula H., Chaouki J., Belmabkhout Y., Zaabout A. (2024). Comparative Review of Direct Air Capture Technologies: From Technical, Commercial, Economic, and Environmental Aspects. Chem. Eng. J..

[133] McQueen N., Gomes K.V., McCormick C., Blumanthal K., Pisciotta M., Wilcox J. (2021). A Review of Direct Air Capture (DAC): Scaling up Commercial Technologies and Innovating for the Future. Prog. Energy.

[134] Min Y.J., Kim J., Jones C.W., Realff M.J. (2024). Model-Based Energy and Cost Analysis of Direct Air Capture Using EPTFE-Based Laminate-Structured Gas-Solid Contactors. ACS Sustain. Chem. Eng..

[135] Eke V., Sahu T., Ghuman K.K., Freire-Gormaly M., O’Brien P.G. (2025). A Comprehensive Review of Life Cycle Assessments of Direct Air Capture and Carbon Dioxide Storage. Sustain. Prod. Consum..

[136] Chen S.F. (2025). Energy and Water Use for DAC: Near-Term Impacts on Resource Availability.

[137] Li S., Zhang Z. (2024). Prospects for Direct Air Capture. Innov. Energy.

[138] Dong H., Wang T., Wang X., Liu F., Hou C., Wang Z., Liu W., Fu L., Gao X. (2023). Humidity Sensitivity Reducing of Moisture Swing Adsorbents by Hydrophobic Carrier Doping for CO_2_ Direct Air Capture. Chem. Eng. J..

[139] Lin M., Ehret C., Hamelers H.V.M., Heijne A., Kuntke P. (2024). Energy Efficient Carbon Capture through Electrochemical PH Swing Regeneration of Amine Solution. ACS Sustain. Chem. Eng..

[140] Realff M.J., Andrus J., Wilcox J. (2021). A Fundamentals-Based Approach for Scaleup of DAC Technology.

[141] Suárez-García S., Nicotera I., Ruiz-Molina D., Simari C. (2023). A mussel-inspired coating for cost-effective and environmentally friendly CO_2_ capture. Chem. Eng. J..

[142] Keith D.W. (2009). Why Capture CO_2_ from the Atmosphere?. Science.

[143] Jiang L., Yong J., Xie R., Xie P., Zhang X., Chen Z., Bao Z. (2023). Screening, Preparation, and Prototyping of Metal-Organic Frameworks for Adsorptive Carbon Capture under Humid Conditions. SusMat.

[144] Goeppert A., Czaun M., Jones J.P., Surya Prakash G.K., Olah G.A. (2014). Recycling of Carbon Dioxide to Methanol and Derived Products-Closing the Loop. Chem. Soc. Rev..

[145] Holmes H.E., Ghosh S., Li C., Kalyanaraman J., Realff M.J., Weston S.C., Lively R.P. (2023). Optimum Relative Humidity Enhances CO_2_ Uptake in Diamine-Appended M2 (Dobpdc). Chem. Eng. J..

[146] Policicchio A., Conte G., Agostino R.G., Caputo P., Oliviero Rossi C., Godbert N., Nicotera I., Simari C. (2022). Hexagonal Mesoporous Silica for carbon capture: Unrevealing CO_2_ microscopic dynamics by Nuclear Magnetic Resonance. J. CO_2_ Util..

[147] Holmes H.E., Banerjee S., Vallace A., Lively R.P., Jones C.W., Realff M.J. (2024). Tuning Sorbent Properties to Reduce the Cost of Direct Air Capture. Energy Environ. Sci..

[148] Lieber A.R., Boone P., He Y., Steckel J.A., Rosi N.L., Wilmer C.E., Hornbostel K.M. (2023). Parametric Simulations of Hierarchical Core–Shell MOF Materials for Direct Air Capture. Sep. Purif. Technol..

[149] Turakulov Z., Kamolov A., Norkobilov A., Variny M., Díaz-Sainz G., Gómez-Coma L., Fallanza M. (2024). Assessing Various CO_2_ Utilization Technologies: A Brief Comparative Review. J. Chem. Technol. Biotechnol..

[150] Zolfaghari Z., Aslani A., Moshari A., Malekli M. (2022). Direct Air Capture from Demonstration to Commercialization Stage: A Bibliometric Analysis. Int. J. Energy Res..

[151] Lackner K.S., Azarabadi H. (2021). Buying down the Cost of Direct Air Capture. Ind. Eng. Chem. Res..

[152] Terlouw T., Bauer C., Rosa L., Mazzotti M. (2021). Life Cycle Assessment of Carbon Dioxide Removal Technologies: A Critical Review. Energy Environ. Sci..

[153] Ozden A., Luo M., Lum Y. (2025). Point-Source Carbon Capture and Direct Air Capture—A Technology Overview. Chem. Eng. J..

[154] McLaughlin H., Littlefield A.A., Menefee M., Kinzer A., Hull T., Sovacool B.K., Bazilian M.D., Kim J., Griffiths S. (2023). Carbon Capture Utilization and Storage in Review: Sociotechnical Implications for a Carbon Reliant World. Renew. Sustain. Energy Rev..

[155] Fridahl M., Lehtveer M. (2018). Bioenergy with Carbon Capture and Storage (BECCS): Global Potential, Investment Preferences, and Deployment Barriers. Energy Res. Soc. Sci..

[156] Fahrudin, Sakti A.D., Komara H.Y., Sumarga E., Choiruddin A., Hendrawan V.S.A., Hati T., Anna Z., Wikantika K. (2024). Optimizing Afforestation and Reforestation Strategies to Enhance Ecosystem Services in Critically Degraded Regions. Trees For. People.

[157] Gailani A., Cooper S., Allen S., Pimm A., Taylor P., Gross R. (2024). Assessing the Potential of Decarbonization Options for Industrial Sectors. Joule.

[158] Farasati Far B., Rabiee N., Iravani S. (2023). Environmental Implications of Metal-Organic Frameworks and MXenes in Biomedical Applications: A Perspective. RSC Adv..

[159] NETL NETL Research Targets Degradation of Sorbents Used in Direct Air Capture. https://www.netl.doe.gov/node/13754.

[160] Kumari S., Gusain M., Lamba B.Y., Kumar S. (2025). A Critical Review on Recent Advancements in Metal-Organic Frameworks for CO_2_ Capture, Storage and Utilization. J. Mater. Chem. A.

[161] Gonzalez-Olmos R., Gutierrez-Ortega A., Sempere J., Nomen R. (2022). Zeolite versus Carbon Adsorbents in Carbon Capture: A Comparison from an Operational and Life Cycle Perspective. J. CO_2_ Util..

[162] Robertson M., Qian J., Qiang Z. (2024). Polymer Sorbent Design for the Direct Air Capture of CO_2_. ACS Appl. Polym. Mater..

[163] Kim K., Kim D., Na Y., Song Y., Wang J. (2023). A Review of Carbon Mineralization Mechanism during Geological CO_2_ Storage. Heliyon.

[164] Sekizkardes A.K., Wang P., Hoffman J., Budhathoki S., Hopkinson D. (2022). Amine-Functionalized Porous Organic Polymers for Carbon Dioxide Capture. Mater. Adv..

[165] IEA (2025). Direct Air Capture—Energy System.

[166] Hack J., Maeda N., Meier D.M. (2022). Review on CO_2_ Capture Using Amine-Functionalized Materials. ACS Omega.

